# Valtrate, an iridoid compound in *Valeriana*, elicits anti-glioblastoma activity through inhibition of the PDGFRA/MEK/ERK signaling pathway

**DOI:** 10.1186/s12967-023-03984-0

**Published:** 2023-02-24

**Authors:** Xuemeng Liu, Yaotian Hu, Zhiyi Xue, Xun Zhang, Xiaofei Liu, Guowei Liu, Muzi Wen, Anjing Chen, Bin Huang, Xingang Li, Ning Yang, Jian Wang

**Affiliations:** 1 Department of Neurosurgery, Cheeloo College of Medicine and Institute of Brain and Brain-Inspired Science, Qilu Hospital, Shandong University, Jinan, 250012 China; 2 Jinan Microecological Biomedicine Shandong Laboratory and Shandong Key Laboratory of Brain Function Remodeling, Jinan, 250117 China; 3 School of Public Health, Southern Medical University, Foushan, 528000 China; 4 Department of Epidemiology and Health Statistics, School of Public Health, Shandong University, Jinan, 250012 China; 5 Department of Biomedicine, University of Bergen, Jonas Lies Vei 91, 5009 Bergen, Norway

**Keywords:** Valtrate, Glioblastoma, Mitochondrial apoptosis, Epithelial mesenchymal transition, Invasion and migration, PDGFRA, MEK, ERK signaling pathway

## Abstract

**Background:**

Valtrate, a natural compound isolated from the root of *Valeriana*, exhibits antitumor activity in many cancers through different mechanisms. However, its efficacy for the treatment of glioblastoma (GBM), a tumor type with a poor prognosis, has not yet been rigorously investigated.

**Methods:**

GBM cell lines were treated with valtrate and CCK-8, colony formation and EdU assays, flow cytometry, and transwell, 3D tumor spheroid invasion and GBM-brain organoid co-culture invasion assays were performed to assess properties of proliferation, viability, apoptosis and invasion/migration. RNA sequencing analysis on valtrate-treated cells was performed to identify putative target genes underlying the antitumor activity of the drug in GBM cells. Western blot analysis, immunofluorescence and immunohistochemistry were performed to evaluate protein levels in valtrate-treated cell lines and in samples obtained from orthotopic xenografts. A specific activator of extracellular signal-regulated kinase (ERK) was used to identify the pathways mediating the effect.

**Results:**

Valtrate significantly inhibited the proliferation of GBM cells in vitro by inducing mitochondrial apoptosis and suppressed invasion and migration of GBM cells by inhibiting levels of proteins associated with epithelial mesenchymal transition (EMT). RNA sequencing analysis of valtrate-treated GBM cells revealed platelet-derived growth factor receptor A (PDGFRA) as a potential target downregulated by the drug. Analysis of PDGFRA protein and downstream mediators demonstrated that valtrate inhibited PDGFRA/MEK/ERK signaling. Finally, treatment of tumor-bearing nude mice with valtrate led to decreased tumor volume (fivefold difference at day 28) and enhanced survival (day 27 vs day 36, control vs valtrate-treated) relative to controls.

**Conclusions:**

Taken together, our study demonstrated that the natural product valtrate elicits antitumor activity in GBM cells through targeting PDGFRA and thus provides a candidate therapeutic compound for the treatment of GBM.

**Supplementary Information:**

The online version contains supplementary material available at 10.1186/s12967-023-03984-0.

## Introduction

Glioblastoma (GBM) is the most common and aggressive primary brain tumor in humans. Despite significant advances in the molecular understanding of the development of GBM in recent years, the treatment of the disease remains challenging. The current standard of care for GBM is still maximal safe resection followed by chemoradiation therapy, and the current first-line clinical treatment is mainly temozolomide. Nevertheless, postoperative GBM recurrence and chemotherapeutic resistance remain as the major challenges in the clinical management of these patients [1, 2]. Therefore, new drugs that are effective in the treatment of GBM are urgently needed.

The search for active ingredients from natural products for the prevention and treatment of malignant tumors is one of the current focuses of antitumor drug development [3]. Valtrate, extracted from the root of *Valeriana*, has been traditionally used to treat various conditions, including nervous, sleep and mental health disorders [4–6]. In recent years, studies have focused on the antitumor effects of valtrate, and reported antitumor activity in the treatment of breast, pancreatic, ovarian and lung cancers. Valtrate was reported to induce apoptosis and G2/M cell cycle arrest in pancreatic cancer cells through direct inhibition of STAT3 activity [7]. The molecule was also shown to cause G2/M cell cycle arrest, apoptosis, and inhibition of cell migration in breast cancer [8]. Moreover, treatment with IVHD-valtrate, another derivative from *Valeriana*, also arrested ovarian cancer cells in the G2/M phase and induced apoptosis [9]. However, the antitumor activity of valtrate and its associated mechanisms of action have yet to be rigorously investigated in GBM.

Our team has long been devoted to research into the anti-GBM properties of herbal medicine, and has reported the roles and related mechanisms of compounds such as flavokawain B, galangin and matrine in the treatment of GBM [10–12]. In the present study, we provide evidence that valtrate inhibits GBM proliferation in vitro and in an orthotopic xenograft model in nude mice. In addition, we perform RNA sequencing analysis to identify a putative target downregulated by valtrate, platelet-derived growth factor receptor A (PDGFRA), and illuminate downstream events coordinating apoptosis and inhibiting migration of GBM cells. These results highlight valtrate as a promising candidate antitumor chemotherapeutic drug in the treatment of GBM.

## Materials and methods

### Ethics statement

Male nude mice (age, 4 weeks; weight, 16 to 20 g) were purchased from the Nanjing Biomedical Research Institute of Nanjing University (Nanjing, China). All animal experiments were approved by the Institutional Animal Care and Use Committee (IACUC) of Shandong University (approval No.: DWLL-2021-104).

### Cell lines and cultures

Human GBM cell lines U251, LN229 and A172 were purchased from the Chinese Academy of Sciences Cell Bank (Shanghai, China). Normal human astrocyte (NHA), green fluorescent protein (GFP)-luciferase-stable U251 (U251^luci^), GFP-luciferase-stable LN229 (LN229^luci^), primary GBM#P3 and glioma stem cells (GSCS) BG5 were obtained from the University of Bergen (Bergen, Norway). GBM cell lines were cultured in Dulbecco’s Modified Eagle Medium (DMEM; Thermo Fisher Scientific; Waltham, MA, USA) supplemented with 10% fetal bovine serum (FBS; Thermo Fisher Scientific) and 1% penicillin/streptomycin (BioNordika; Oslo, Norway). Cells (GBM#P3 and GSCS BG5) were cultured in serum-free Neurobasal Medium (NBM; ThermoFisher Scientific) supplemented with 2% B27 (ThermoFisher Scientific), 1% L-glutamine (BioNordika, 1% penicillin/streptomycin (BioNordika), epidermal growth factor (20 ng/mL; Peprotech; Rocky Hill, NJ, USA), and basic fibroblast growth factor (20 ng/mL; Peprotech). Cells were incubated at 37 ℃ in 5% CO2 in a humidified chamber.

### Cell viability and proliferation assays

Cell viability was assessed with the Cell Counting Kit-8 assay (CCK-8; Dojindo, Kumamoto, Japan). GBM cells (cell lines, 5 × 10^3^ cells/well; GBM#P3 and BG5, 1 × 10^4^ cells/well) were seeded into 96-well plates and cultured at 37 ℃. After about 24 h, the medium was replaced with 100 μL of fresh culture medium containing different concentrations of valtrate or dimethylsulfoxide (DMSO). After 24, 48 and 72 h of treatment, 10 μL of CCK-8 solution was added to each well, and the cells were cultured for 1 h at 37 °C. Cell viability was quantified on the EnSight Multimode Plate Reader (PerkinElmer; Singapore) at 450 nm. Proliferation was assessed using the EdU incorporation assay according to the manufacturer’s protocol (Ribobio, C103103; Guangzhou, China). In the extreme limiting dilution assay, BG5 cells were placed in a 96-well plate at a density of 1 to 50 cells/well with six replicates for each concentration of drug. After 10 days, the numbers of tumorspheres in each well were recorded. Assays were performed in three independent experiments.

### Cellular morphology analysis

U251 and LN229 cells were seeded into 6-well plates and treated with valtrate at different concentrations for 48 h. The cellular morphology was observed under phase contrast microscopy. Cells were stained with the fluorescent DNA binding dye Hoechst 33342 (Beyotime Institute of Biotechnology; Shanghai, China) for 15 min in the dark at room temperature, and the nuclear morphology was observed under fluorescence microscopy.

### Colony formation assay

GBM cells (U251 and LN229) were seeded into 6-well plates (800 cells/well) containing 2 mL of complete medium. After 24 h, the medium was replaced with complete medium containing different concentrations of valtrate or DMSO (vehicle control). After 7 days, the medium was replaced with fresh medium, and the cells were cultured for another 7 days. The cells were fixed with 4% paraformaldehyde for 15 min and stained with crystal violet for 30 min. The cells were rinsed with 500 μL of PBS three times, and the colonies were counted (> 50 cells).

### Cell apoptosis assay

Both adherent and floating cells were harvested, rinsed in PBS twice, resuspended in 1 × binding buffer and then stained with annexin V-FITC and PI according to the manufacturer’s protocol. Samples were analyzed on a flow cytometer (ACEA Biosciences; San Diego, CA, USA), and the results were analyzed using the software Flowjo (Tree Star; Ashland, OR, USA).

### Measurement of mitochondrial function, membrane potential and morphological changes of mitochondria

U251 and LN229 cells were seeded into a 24-well plate, treated with valtrate for 48 h and then stained with the Mitochondrial Membrane Potential and Apoptosis Detection Kit with Mito-Tracker Red CMXRos and Annexin V-FITC (Beyotime Institute of Biotechnology) at 37 ℃ for 30 min according to the manufacturer’s instructions. The samples were evaluated under florescence microscopy to detect the mitochondrial membrane potential. Mito-Tracker Green (Beyotime Institute of Biotechnology) was used as a probe to assess mitochondrial function. U251 and LN229 cells were seeded into a 24-well plate, treated with valtrate for 48 h and then stained with Mito-Tracker Green at 37 ℃ for 30 min in the dark. Samples were observed under fluorescence microscopy. Transmission electron microscopy (TEM) was used to assess morphological changes in mitochondria.

### Cell invasion and migration assays

For the 3D tumor spheroid invasion assay, U251 and GBM#P3 tumor spheroids were first generated in 3D Culture Qualified 96-well spheroid formation plates (Trevigen; Minneapolis, MN). Invasion matrix (Trevigen) with different concentrations of valtrate was added to the wells, and the invasion distance of cells into the matrix away from the sphere was measured every day. For the GBM-brain organoid co-culture invasion assay, 18-day fetal mice brain organoids were prepared as described in our previous work [13] and then co-cultured with U251 and GBM#P3 tumor spheres generated through culture of cells in 3D Culture Qualified 96-well spheroid formation plates (Trevigen) for approximately 4 days. The rate of tumorsphere invasion into the brain organoids at different drug concentrations was assessed under confocal microscopy (Leica TCS SP8, Leica; Wetzlar, Germany). For transwell assays, U251 and LN229 were seeded into wells of a 6-well plate, cultured for 24 h and then treated with valtrate at different concentrations. After 48 h, the cells were harvested and seeded (20,000 cells) into the upper chamber of a transwell apparatus (Corning; Sigma-Aldrich; St. Louis, MO, USA) in 200 μL of DMEM without FBS, and 600 μL of medium containing 10% FBS was added to the lower chamber. After incubation in the chamber at 37 °C in 5% CO2 for 24 h, the cells were fixed with 4% paraformaldehyde for 15 min and stained with crystal violet for 30 min. Images of migrated cells were acquired with an inverted microscope.

### Western blot analysis

U251, LN229 and GBM#P3 cells were seeded into 6-well plates for 24 h, treated with valtrate for 48 h, and then collected and lysed. Protein concentrations were determined with the BCA assay kit (Beyotime Institute of Biotechnology). Proteins (20 µg/lane) were separated on 10% SDS‑PAGE and transferred to PVDF membranes (GVW2932A, 0.22 μm, Millipore Sigma; Burlington, MA, USA). Membranes were blocked in 5% skim milk in Tris-buffered saline containing 0.1% Tween-20 for 2 h, incubated with primary antibodies overnight at 4 °C, rinsed three times with TBS supplemented with 0.1% Tween‑20 (TBST), incubated with secondary antibodies (1:5000) at room temperature for 1 h, and rinsed three times with TBST. Finally, membranes were exposed to ECL reagent and visualized on the Chemiluminescence Imager (Bio-Rad; Hercules, CA, USA).

### Immunofluorescence (IF) staining

U251-GFP and LN229-GFP cells were cultured in 24-well plates with valtrate at different concentrations for 48 h, fixed with 4% paraformaldehyde at room temperature for 15 min, and treated with 0.5% Triton X-100 in PBS at room temperature for 15 min. For detection of PDGFRA protein, cells were blocked with 5% bovine serum albumin (BSA) in PBS for 1 h at room temperature, and incubated first with PDGFRA antibody overnight at 4 °C and subsequently with secondary antibodies (1:200) for 1 h. Nuclei were stained with 2-(4-Amidinophenyl)-6-indolecarbamidine dihydrochloride (DAPI) for 5 min at room temperature. Images were acquired under confocal microscopy.

### Immunohistochemistry (IHC) and hematoxylin and eosin staining (H&E) staining

Sections of paraffin-embedded tumors/tissue were dewaxed and hydrated, and antigen retrieval was performed in 1 × EDTA. Endogenous peroxidase activity was quenched in sections through incubation with 3% H2O2 at room temperature for 15 min. The sections were blocked with serum for 30 min, incubated with the primary antibody (Ki67, PDGFRA, p-MEK1/2, p-ERK1/2, Bax and Bcl2, 1:200, diluted in phosphate-buffered saline (PBS)) overnight at 4 °C, rinsed with PBS, and finally incubated with secondary antibody at room temperature for 1 h. For detection, 100 μL of DAB staining solution was added to each sample and images were acquired with an inverted microscope. H&E staining was used for histological examination of the main organs of tumor-bearing mice treated with valtrate. Images were acquired under bright field microscopy (Olympus; Tokyo, Japan).

### TUNEL assay

The Colorimetric TUNEL Apoptosis Assay Kit (Beyotime Institute of Biotechnology) was used to evaluate apoptosis in sections from tissues exposed to valtrate. Tumor sections were dewaxed, hydrated and treated with 20 μg/mL proteinase K for 30 min at 37 °C. The TUNEL assay was performed according to the manufacturer’s instructions.

### RNA sequencing and bioinformatic analysis

RNA-Seq libraries were prepared as described in our previous report [14] to perform transcriptome profiling. RNA-Seq data were used to identify differentially expressed genes and associated signaling pathways in GBM cells treated with valtrate compared to controls. Genes were selected with basal mRNA expression levels of (the mRNA expression in controls) > 1000 in U251 cells and > 3000 in GBM#P3 cells for co-expression differential analysis. Gene ontology (GO) and Kyoto Encyclopedia of Genes and Genomes (KEGG) pathways enrichment analyses were performed based on the identified differentially expressed genes (P value ≤ 0.05, |Log2FC|≥ 1). Differential expression of *PDGFRA* in GBM was analyzed with the online analysis site GEPIA2 (http://gepia2.cancer-pku.cn/#analysis), and R language was used to obtain Kaplan–Meier (KM) curves for patients with high or low *PDGFRA* expressing GBMs.

### Transient transfection of the PDGFRA expression construct

The PDGFRA expression construct was generated based on the sequence available in GenBank (NM_006206.4) and standard molecular biological methods. The sequence of the final construct was confirmed through full-length sequencing with the following primers: 5ʹ-CAGGTGTCCACTCCCAGGTCCAAG-3ʹ and 5ʹ-GGCAACTAGAAGG CACAGTCGAGG-3ʹ. Cells (2.0 × 10^5^) were seeded into 6-well plates 24 h before transfection. A mixture of transfection reagent and DNA was determined based on the following specifications for the Micropoly-transfecter^™^ cell reagent (Micropoly; Nantong, China): 2 μg DNA: 2 μL reagent per well for 6-well plates. The mixture was incubated at room temperature for 10 min, and then added to the cells cultured in complete medium. Western blot analysis was used to evaluate the transfection efficiency.

### Intracranial xenograft model

GBM#P3^luci^ cells were implanted into the right striatum of nude mice to assess antitumor activity in vivo in an intracranial xenograft model, and implanted mice were randomized into treatment and control groups (n = 10/group). Valtrate was dissolved in the vehicle (50% PBS, 40% Kolliphor/ethylene glycol, and 10% DMSO) based on a previous report [7]. Mice were intraperitoneally injected with either valtrate (15 mg/kg) or the same vehicle volume every day. Body weight was measured every 3 days, and tumor size was monitored every 3 days up to 28 days with a cooled charge coupled device camera (IVIS-200, Xenogen; Alameda, CA, USA). The bioluminescence values of tumors were quantitated with the Living Image 2.5 software package (Xenogen). The brain and main organs were removed, fixed, embedded in paraffin and sectioned for IHC and H&E staining.

### Statistical analysis

Statistical analysis was performed using GraphPad Prism 8.0 Software (Boston, MA, USA). All data are expressed as the mean ± standard deviation (SD) from three replicate experiments. The significance of differences between groups was determined using a Student’s *t* test. A p-value of < 0.05 was considered statistically significant.

## Results

### Valtrate suppresses the proliferation of human GBM cells

**Fig. 1:**
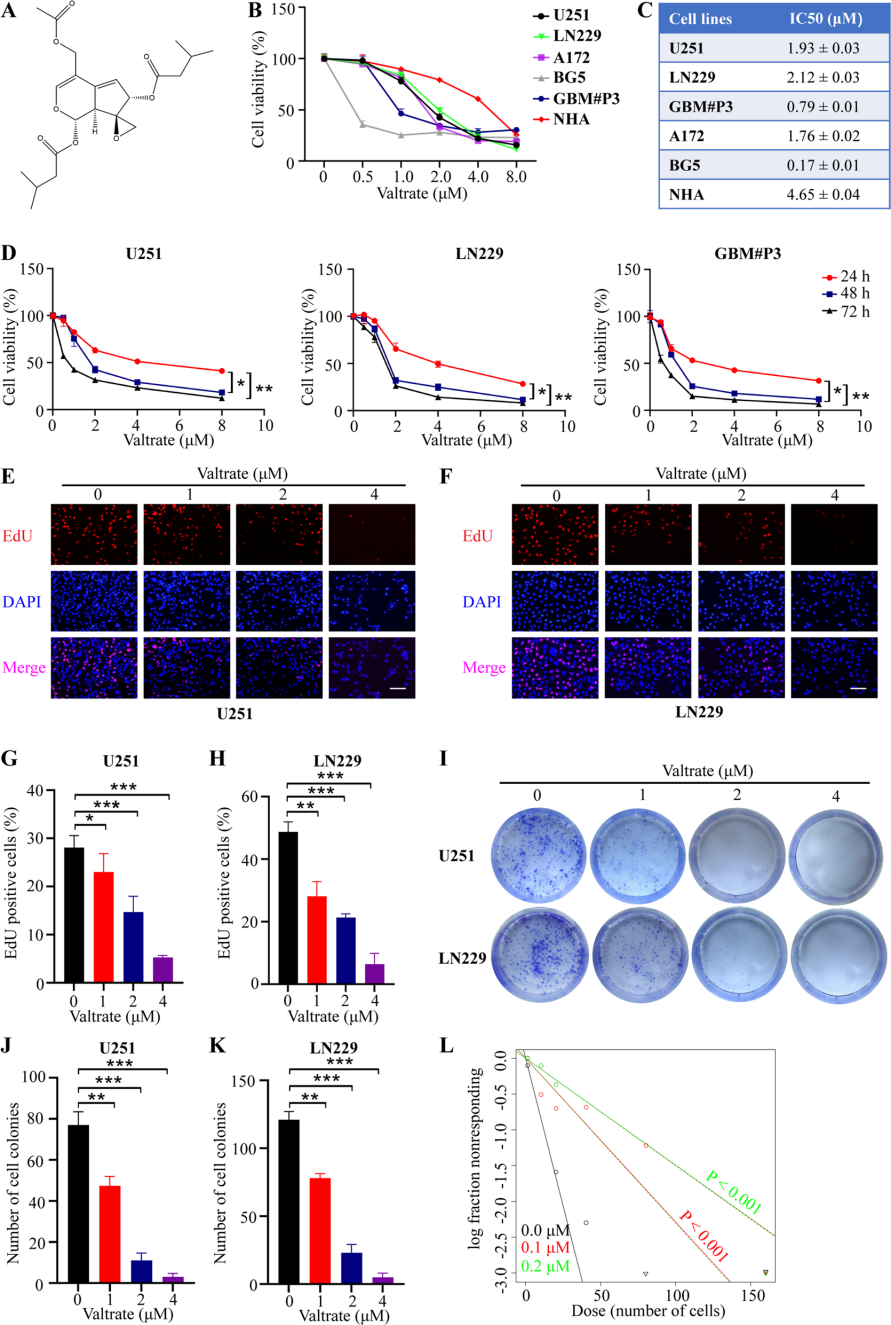
Valtrate inhibits cell proliferation in GBM cells. **A** Illustration of the chemical structure of valtrate derived in ChemDraw 20.0. **B** Cell viability determined with the CCK-8 assay for U251, LN229, A172, GBM#P3, BG5 and NHA treated with different concentrations of valtrate for 48 h. **C** IC50 values of valtrate for U251, LN229, A172, GBM#P3, BG5 and NHA. **D** Cell viability of U251, LN229 and GBM#P3 with valtrate at different concentrations (U251, LN229: 0, 0.5, 1.0, 2.0, 4.0, and 8.0 μM; GBM#P3: 0, 0.25, 0.5, 1.0, 2.0, and 4.0 μM) and at different time points (24, 48 and 72 h). **E**–**H** Representative images of EdU assays for U251and LN229 treated with different concentrations of valtrate for 48 h and quantitative analysis. The percentage of EdU-positive cells was quantified in three random fields per sample. Scale bar, 50 μm. **I**–**K** Representative images of colony formation assays for U251 and LN229 cells with treatment of valtrate for 14 days and quantitative analysis of colony numbers. **L** Extreme limiting dilution assay performed with BG5. Data are shown as the mean ± SD and the differences between groups were analyzed with the Student’s *t*-test. *p < 0.05, **p < 0.01, ***p < 0.001

To begin to investigate whether valtrate (chemical structure, Fig. 1A) exhibits antitumor activity in GBM, GBM cells, including U251, LN229, A172, GBM#P3, and GSCS BG5, and NHA, were exposed to the small molecule, and proliferation was first assessed in the CCK-8 assay (Fig. 1B). Cell viability of all cell types decreased with increasing concentration of valtrate, and the IC50s at 48 h demonstrated that some GBM cells were more sensitive to the drug than others. NHA were the least sensitive of all cells tested (Fig. 1C, Additional file 1: Figure S1A). The inhibitory effect of valtrate was not only concentration-dependent but also time-dependent (Fig. 1D). The EdU-DNA synthesis assay also demonstrated that increasing valtrate concentration led to increased inhibition of DNA replication in U251 and LN229 cells (Fig. 1E–H). U251 and LN229 cells treated with valtrate also yielded fewer colonies in the colony forming assay, demonstrating that the molecule inhibited proliferation of GBM cells in vitro (Fig. 1I–K).

Finally, we asked whether valtrate suppressed stem cell properties of GBM cells. In tumorsphere formation and extreme limiting dilution assays, valtrate inhibited the formation of tumorspheres. Thus, valtrate inhibited proliferation and the stemness of GBM cells (Fig. 1L, Additional file 1: Figure S1B). All together, these data indicated that valtrate reduced the viability of GBM cells in vitro.

### Valtrate induces mitochondrial apoptosis in GBM cells

**Fig. 2:**
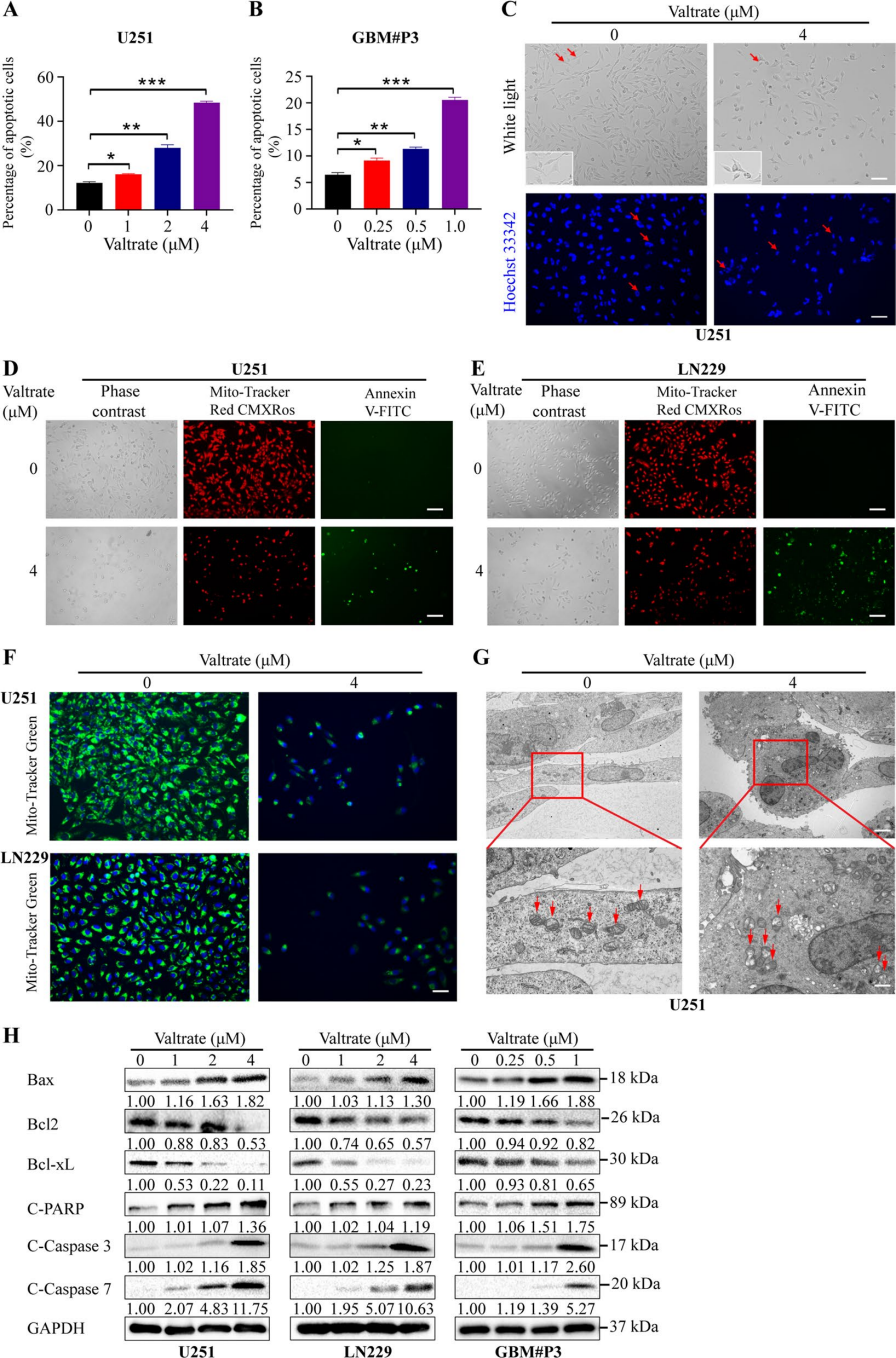
Valtrate promotes apoptosis in GBM cells via the mitochondrial pathway. **A**–**B** Bar graphs representing quantitative analysis of valtrate-induced U251 and GBM#P3 apoptosis assessed with flow cytometry. **C** Representative images of the cellular morphology of valtrate-treated U251 at 48 h, as observed under a phase contrast microscope. Enlarged images in the inset of U251 cells with morphological changes highlighted by the red arrows (upper panel). Fluorescence imaging of valtrate-treated cells stained with Hoechst 33342 to examine nuclear morphology (lower panel). Scale bar, 50 μm. **D**–**E** Representative images of valtrate-treated U251 and LN229 cells stained with Mito-Tracker Red CMXRos and Annexin V-FITC to assess mitochondrial membrane potential and apoptosis. Red fluorescence represents live cells that maintain mitochondrial membrane potential and green fluorescence, cells that have undergone apoptosis or necrosis. Scale bar, 50 μm. **F** Fluorescence images of valtrate-treated U251 and LN229 cells stained with Mito-Tracker Green to assess mitochondrial quality. The green fluorescence of the cells is diminished in valtrate-treated cells, indicating a decrease in mitochondrial mass. Scale bar, 50 μm. **G** Transmission electron microscopy to assess the mitochondrial ultrastructure of valtrate-treated U251 cells relative to controls. Scale bar, 5.0 μm; scale of the local enlargement, 2.0 μm. The red arrows highlight the mitochondria. **H** Western blot for the detection of the expression levels of apoptosis-related proteins in U251, LN229 and GBM#P3 cells treated with different concentrations of valtrate. Data are shown as the mean ± SD and the differences between groups were analyzed with the Student’s *t*-test. *p < 0.05, **p < 0.01, ***p < 0.001

To investigate the mechanisms underlying valtrate‑mediated growth inhibition, valtrate-treated GBM cells were first examined by flow cytometry for apoptosis. The percentage of annexin-V-FITC and PI positive cells increased with valtrate treatment in a dose-dependent manner (Fig. 2A–B, Additional file 1: Figure S2A–B). Morphological changes were also visible in cells and nuclei, and were consistent with cells undergoing apoptosis. Valtrate-treated cells appeared to have a round and shrunken shape (Fig. 2C, Additional file 1: Figure S2C, upper panel), and the nuclei, stained with Hoechst 33,342, appeared concentrated and disrupted (Fig. 2C, Additional file 1: Figure S2C, lower panel). In contrast, the nuclei in the control cells were morphologically normal and uniformly distributed (Fig. 2C, Additional file 1: Figure S2C, lower panel).

The red fluorescence of the fluorescent stain Mito-Tracker Red CMXRos gradually diminished, or was negative in cells treated with 4 μM valtrate (Fig. 2D–E), which was consistent with decreased mitochondrial membrane potential and apoptosis. In contrast, control cells exhibited bright red fluorescence. Mito-Tracker Green, a mitochondrial green fluorescent probe, is used for mitochondria-specific fluorescence staining in living cells and is not dependent on the mitochondrial membrane potential. In U251 and LN229 cells, green fluorescence was reduced with valtrate treatment, indicating that valtrate caused a decrease in the quality of mitochondria in GBM cells (Fig. 2F).

We further investigated the morphology of mitochondria in U251 and LN229 cells using transmission electron microscopy. In valtrate-treated U251 and LN229 cells (4 μM), the membranes of mitochondria were disrupted, and the cristae appeared to be broken and dissolved, or had disappeared. The central area of the mitochondria showed clear cristae density, and the mitochondria appeared swollen and damaged (Fig. 2G and Additional file 1: Figure S2D). In contrast, the cell structures were intact in the control cells, exhibiting normal cell membrane structures and clear and intact connections in the cristae. These results indicated that valtrate disrupted mitochondrial structures and induced mitochondrial damage in U251 and LN229 cells (Fig. 2G, Additional file 1: Figure S2D).

We also examined the levels of proteins associated with apoptosis in treated cells on western blot. Bcl-2-associated x protein (Bax), a pro-apoptotic protein located on the mitochondrial membrane, was upregulated, while B-cell lymphoma 2 (Bcl2) and Bcl-xL, two anti-apoptotic proteins, were decreased (Fig. 2H). In addition, the levels of ATP were significantly decreased in U251, LN229 and GBM#P3 cells after treatment with valtrate for 48 h compared to the control group. This result was consistent with cells undergoing apoptosis as a decrease in ATP levels is another indicator of a decrease or impairment of cellular mitochondrial function (Additional file 1: Figure S2E). These results together demonstrated that valtrate triggered mitochondrial apoptosis in GBM cells.

### Valtrate inhibits migration and invasion of GBM cells through suppression of EMT

**Fig. 3:**
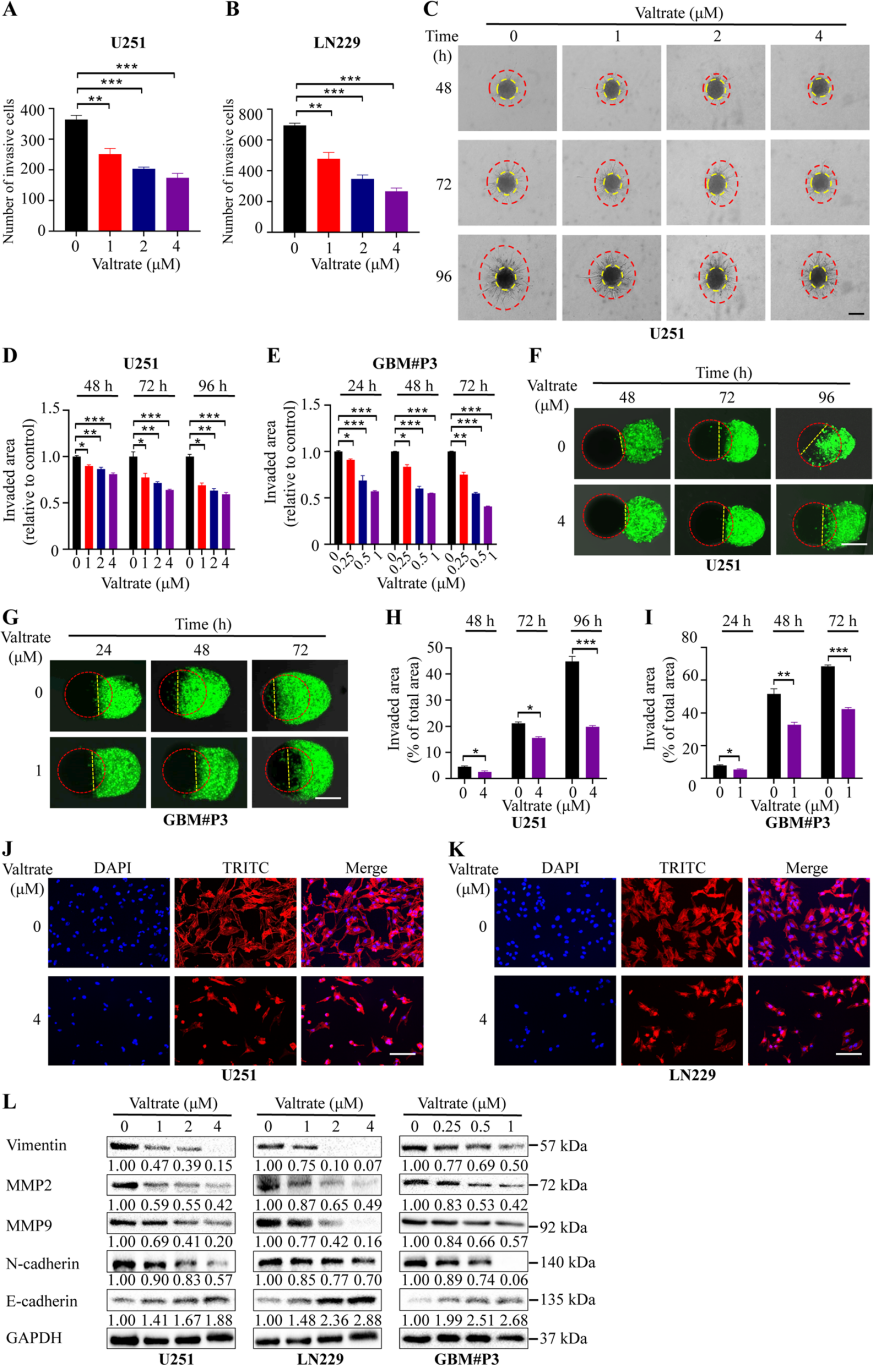
Valtrate inhibits migration and invasion of GBM cells by suppressing EMT. **A**–**B** Quantitative analysis of the Transwell assay. **C**–**E** Representative images and quantification of 3D invasion assays for U251 and GBM#P3 cells treated with different concentrations of valtrate at different time points. Scale bar, 200 μm. **F**–**I** Representative images of the brain organoid-tumor cell spheroid coculture invasion assay for U251 and GBM#P3 cells treated with valtrate and the quantitative analysis. The red dotted line represents the brain organoid, and the yellow dotted line represents the area invaded by the GBM spheroid at the indicated concentrations of valtrate. Scale bar, 100 μm. **J**–**K** FITC-phalloidin staining to highlight the morphology of the cytoskeleton in valtrate-treated U251 and LN229 cells compared to controls. Scale bar, 100 μm. **L** Western blot to detect expression of EMT related molecules in U251, LN229 and GBM#P3 after treatment with valtrate for 48 h. Data are shown as the mean ± SD and the differences between groups were analyzed with the Student’s *t*-test. *p < 0.05, **p < 0.01, ***p < 0.001

Infiltrative growth is one of the hallmark features of GBM. We therefore explored whether valtrate inhibited the invasion and migration ability of GBM cells in transwell and 3D spheroid invasion assays. The number of invasive cells in the transwell assay was significantly decreased for valtrate-treated cells compared to control cells (Fig. 3A–B, Additional file 1: Figure S3A–B). Furthermore, outward invasion of valtrate-treated GBM cells into the matrix from spheres was inhibited in the 3D spheroid invasion assay (Fig. 3C–E, Additional file 1: Figure S3C). Finally, in the GBM spheroid and brain organoid coculture assay, the area of the valtrate-treated tumor cells invading into the brain organoid compared to the control group was also reduced (Fig. 3F–I).

Next, we performed phalloidin immunofluorescence staining to examine the morphological features of the actin filaments in valtrate-treated cells. While the control cells exhibited a branching pattern typical of actin filaments in invasive cells, the valtrate-treated cells showed a different morphology of the actin cytoskeleton, which featured few branches and a tendency to be round (Fig. 3J–K). Such changes in actin branching and loss of cell–cell contact are characteristics of the changes in cell morphology that occur during EMT.

Protein levels of EMT markers were also examined by western blot in U251, LN229 and GBM#P3 cells treated with valtrate. The expression levels of EMT-related proteins, such as vimentin, MMP9 and N-cadherin, were significantly downregulated, whereas E-cadherin was upregulated in valtrate-treated cells (Fig. 3L). These results suggested that valtrate inhibits the invasion and migration of GBM cells by suppressing EMT.

### PDGFRA is a potential target of valtrate

**Fig. 4:**
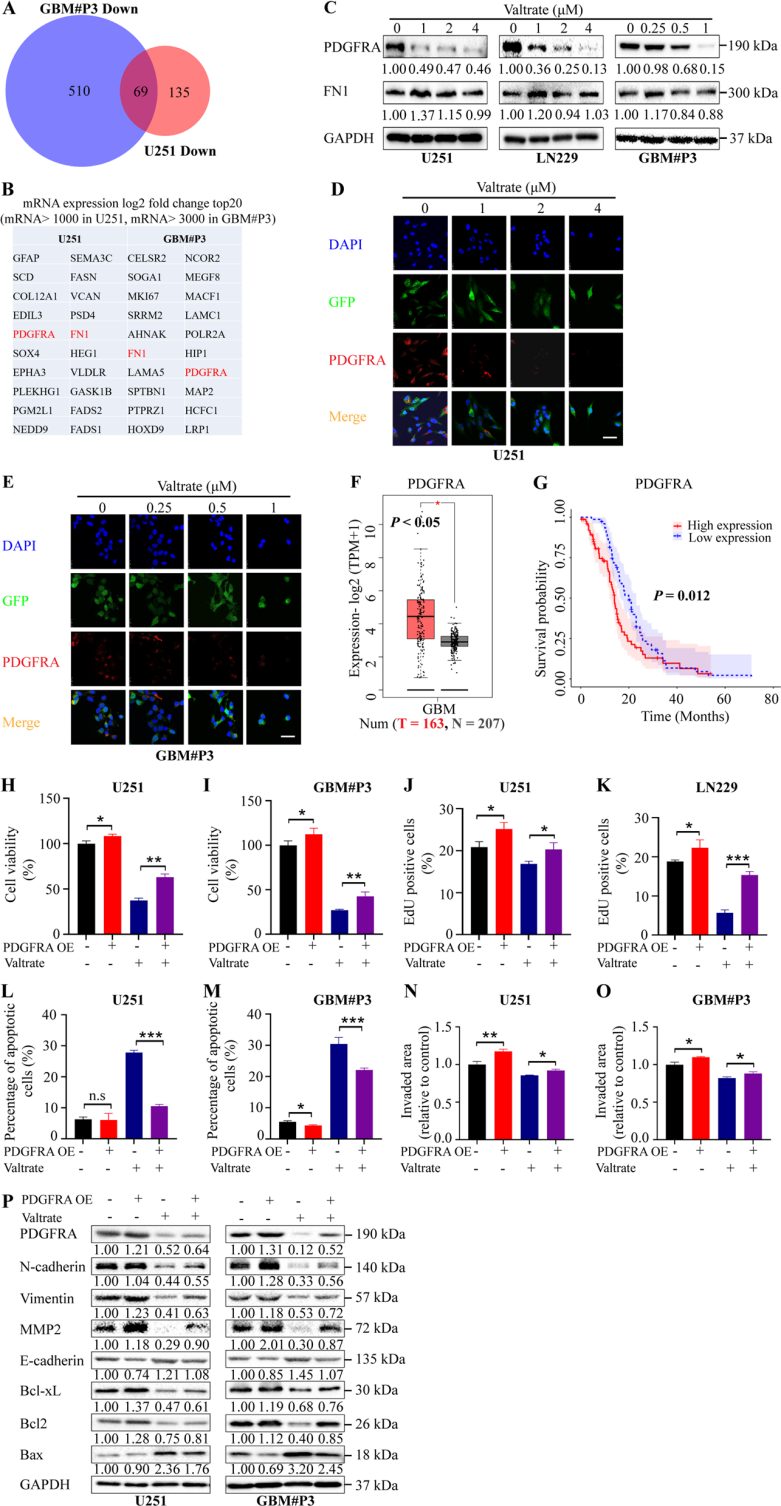
PDGFRA is a potential target downregulated by valtrate in GBM cells. **A** RNA-Seq data analysis showing the 69 intersecting downregulated genes in valtrate-treated U251 and GBM#P3 for 48 h. **B** Top 20 genes out of the 69 genes with the greatest log2 fold change in mRNA expression levels (basal mRNA expression level in controls of  > 1000 in U251, and  > 3000 in GBM#P3). Intersecting genes (out of the top 20) *PDGFRA* and *FN1* are highlighted in red. **C** Western blot to determine expression levels of PDGFRA, FN1 and GAPDH (protein loading control) in U251, LN229 and GBM#P3 cells treated with DMSO or valtrate at the indicated concentrations for 48 h. **D**–**E** Representative images of immunofluorescence staining for PDGFRA in U251- and GBM#P3-GFP cells after treatment with the indicated concentration of valtrate for 48 h. Nuclei are stained with DAPI; GFP highlights the cells. Scale bar, 200 μm. **F** Analysis of expression levels of *PDGFRA* mRNA in normal brain tissue samples (N = 207) and GBM samples (T = 163) from the public database TCGA. *p < 0.05. **G** Kaplan–Meier analysis of overall survival for GBM patients with *PDGFRA*^high/low^ GBM. *PDGFRA*^high^ GBM, *PDGFRA* > median expression; *PDGFRA*.^low^ GBM, *PDGFRA* < median expression. **H**–**I** Cell viability of U251- and GBM#P3-PDGFRA-OE under the conditions indicated, as determined with the CCK-8 assay. **J**–**K** Analysis of EdU assays for U251- and GBM#P3-PDGFRA-OE cells, showing ectopic expression of PDGFRA rescues the inhibitory effect of valtrate on the proliferation of U251 and GBM#P3 cells. **L**–**M** Percentage of apoptotic U251- and GBM#P3-PDGFRA-OE cells under valtrate treatment relative to controls. **N**–**O** Analysis of 3D spheroid invasion assays for U251- and GBM#P3-PDGFRA-OE cells under valtrate treatment relative to controls. **P** Western blot to determine levels of protein markers of apoptosis, invasion and migration in U251- and GBM#P3-PDGFRA-OE relative to controls under the conditions indicated. Data are shown as the mean ± SD and the differences between groups were analyzed with the Student’s *t*-test. *n.s* none significant, *p < 0.05, **p < 0.01, ***p < 0.001

To identify putative genes regulated by valtrate in GBM cells, we performed RNA sequencing on mRNA isolated from valtrate-treated U251 and GBM#P3 cells and identified differentially expressed genes the two cell lines had in common. Analysis of the RNA-Seq data revealed 69 downregulated genes in common (Fig. 4A). Only two genes, *PDGFRA* and *fibronectin-1* (*FN1*), were found to be among the top 20 most differentially downregulated genes in U251 and GBM#P3 cells (Fig. 4B, Additional file 1: Figure S4A). However, we found that only PDGFRA was significantly downregulated at the protein level in valtrate-treated U251 and GBM#P3 cells (Fig. 4C). Immunofluorescence staining also demonstrated that valtrate suppressed PDGFRA expression in a concentration-dependent manner (Fig. 4D–E).

To investigate the clinical relevance of PDGFRA in GBM, we examined the expression levels of the gene through analysis of the RNA-Seq data from Gene Expression Profiling Interactive Analysis (GEPIA). *PDGFRA* was highly upregulated in GBM compared with normal brain tissue samples (Fig. 4F), and Kaplan–Meier analysis of TCGA and CGGA datasets revealed that patients with *PDGFRA*^high^ GBM exhibited shorter median overall survival relative to patients with *PDGFRA*^low^ GBM (Fig. 4G).

To determine whether increased expression of PDGFRA rescued the growth inhibition caused by valtrate, we transiently transfected PDGFRA expression constructs into GBM cells. Overexpression of PDGFRA reversed the decrease in cell viability and proliferation caused by valtrate as assessed in CCK-8 and EdU assays for U251, LN229 and GBM#P3 cells (Fig. 4H–K, Additional file 1: Figure S4B–C). Valtrate-induced apoptosis was also reduced in treated cells with ectopic expression of PDGFRA relative to treated controls (Fig. 4L–M, S4D). Overexpression of PDGFRA in cells also led to increased invasion and migration of GBM cells in the presence of valtrate (Fig. 4N–O, Additional file 1: Figure S4E). Finally, overexpression of PDGFRA partially decreased apoptosis and reversed the changes in EMT-related markers caused by valtrate (Fig. 4P). These results suggest that valtrate elicits anti-glioblastoma activity by targeting PDGFRA.

### Valtrate negatively regulates the PDGFRA/MEK/ERK signaling pathway in GBM cells

**Fig. 5:**
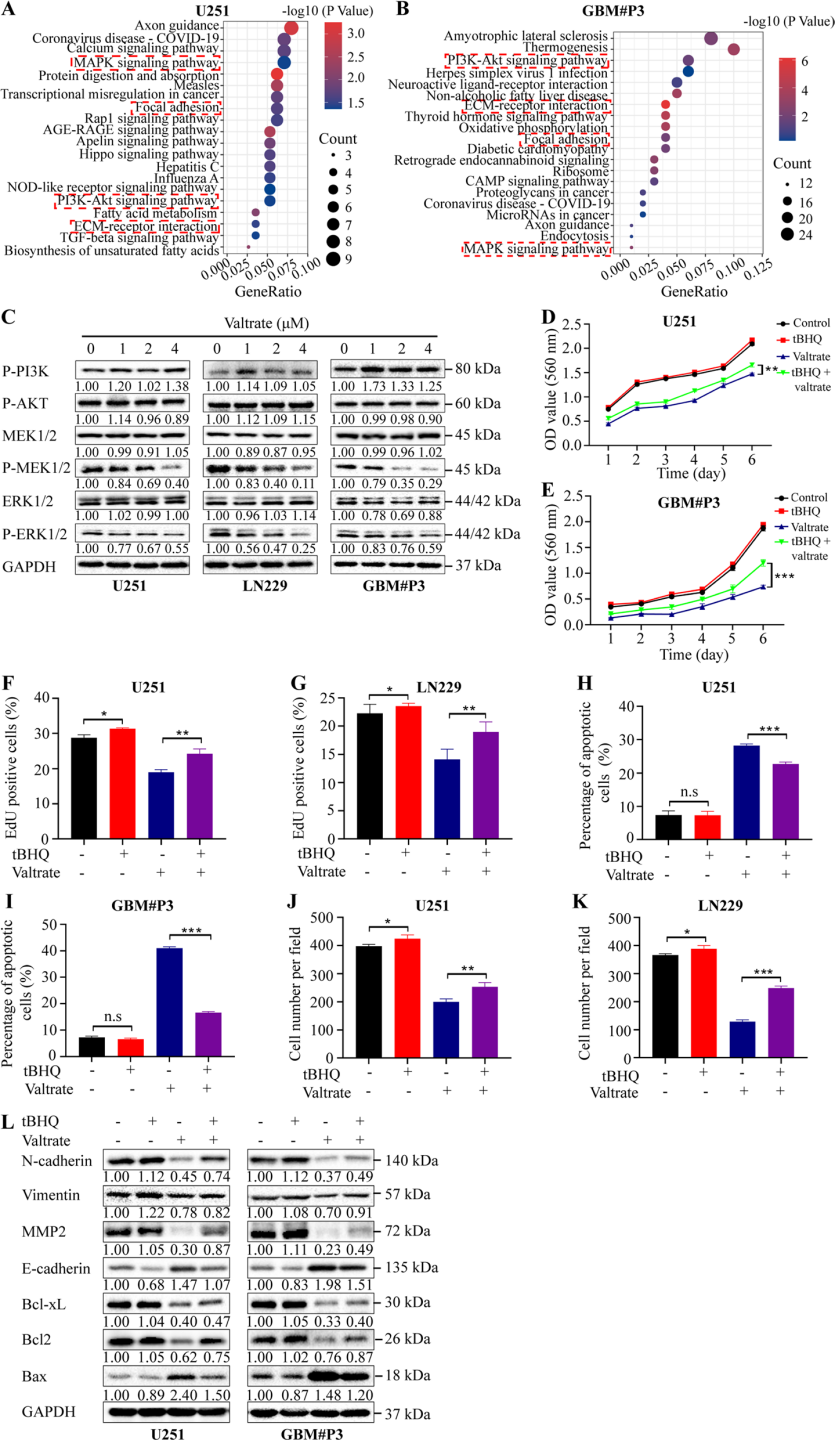
Valtrate elicits anti-glioblastoma activity through inhibition of the PDGFRA/MEK/ERK signaling pathway. **A**–**B** Pathway enrichment analysis of the RNA-Seq data from valtrate-treated U251 and GBM#P3 cells. **C** Western blot to detect levels of proteins involved in PI3K/AKT and MEK/ERK signaling pathways in U251, LN229 and GBM#P3 after treatment with different concentrations of valtrate. **D**–**E** Cell viability determined with the CCK-8 assay for U251 and GBM#P3 cells under valtrate treatment in the presence of the ERK1/2 activator t-butylhydroquinone (tBHQ). U251: 2 μΜ valtrate; GBM#P3: 1 μΜ valtrate; and 50 μM tBHQ. **F**–**G** Quantitative analysis of EdU assays performed on U251 and LN229 under the conditions indicated, under valtrate treatment with or without the activator of ERK tBHQ. U251: 2 μΜ valtrate; LN229: 2 μΜ valtrate; and tBHQ: 50 μM. **H**–**I** Percentage of apoptotic U251 and GBM#P3 cells under the conditions indicated, under valtrate treatment with or without tBHQ. U251: 2 μΜ valtrate, GBM#P3: 1 μΜ valtrate; and tBHQ: 50 μM. **J**–**K** Quantification of transwell cell numbers for U251 and LN229 under the conditions indicated, under valtrate treatment with or without tBHQ. U251: 2 μΜ valtrate; LN229: 2 μΜ valtrate; and tBHQ: 50 μM). **L** Western blot to detect the levels of mitochondrial apoptosis- and EMT-related proteins in U251 and GBM#P3 cells with the valtrate or tBHQ treated. U251: 2 μΜ valtrate; GBM#P3: 1 μΜ valtrate; and tBHQ: 50 μM. Data are shown as the mean ± SD and the differences between groups were analyzed with the Student’s *t*-test. *n.s* none significant, *p < 0.05, **p < 0.01, ***p < 0.001

PDGFRA promotes tumor development by activating downstream signaling kinases, such as extracellular signal-regulated kinase (ERK), AKT and S6 K [15, 16]. Kyoto Encyclopedia of Genes and Genomes (KEGG) analysis of RNA-Seq data showed that differentially expressed genes due to valtrate treatment of GBM cells were associated with cell invasive migration, and mitogen-activated protein kinase (MAPK) and phospatidylinositol-3 kinase (PI3K)-AKT signaling pathways (Fig. 5A and B). Based on previous studies [17, 18] and our own results from antibody arrays [19], PI3K/AKT and MEK/ERK signaling pathways have emerged as two important signaling pathways involved in the development of GBM. Therefore, we further investigated whether phosphorylated PI3K/AKT and MEK/ERK were involved in the inhibition of proliferation, invasion and migration, and the promotion of apoptosis by valtrate in GBM cells. First, we examined the levels of phosphorylated-MEK, -ERK, -PI3K and -AKT in U251, LN229, and GBM#P3 cells after valtrate treatment on western blot. Phosphorylated-MEK (Ser221) and -ERK (Thr202/Tyr204) were both decreased in valtrate-treated cells, while phosphorylated-PI3K (Tyr458) and -AKT (Thr308) remained unchanged in all three GBM cell lines (Fig. 5C). To further investigate the role of the ERK signaling pathway in the treatment of GBM with valtrate, we exposed GBM cells to an activator of ERK, t-butylhydroquinone (tBHQ) (50 μM), and evaluated proliferation and cell viability of U251, LN229 and GBM#P3 treated with valtrate. The presence of tBHQ suppressed valtrate-induced activity, including the decrease in cell viability and proliferation in GBM cells (Fig. 5D–G, Additional file 1: Figure S5A–B) and the promotion of apoptosis (Fig. 5H–I, Additional file 1: Figure S5C). Cell migration and invasion were also enhanced by tBHQ in valtrate-treated GBM cells in vitro (Fig. 5J–K, Additional file 1: Figure S5D).

Finally, tBHQ altered levels of proteins upregulated or downregulated by valtrate indicating that activated ERK directly or indirectly regulated cell invasion/migration and apoptosis-related proteins (Fig. 5L). Taken together, our results indicate that suppressing the PDGFRA/MEK/ERK signaling pathway is a possible mechanism underlying the anti-GBM activity of valtrate.

### Valtrate potently inhibits glioblastoma multiforme xenograft tumor growth in mice

**Fig. 6:**
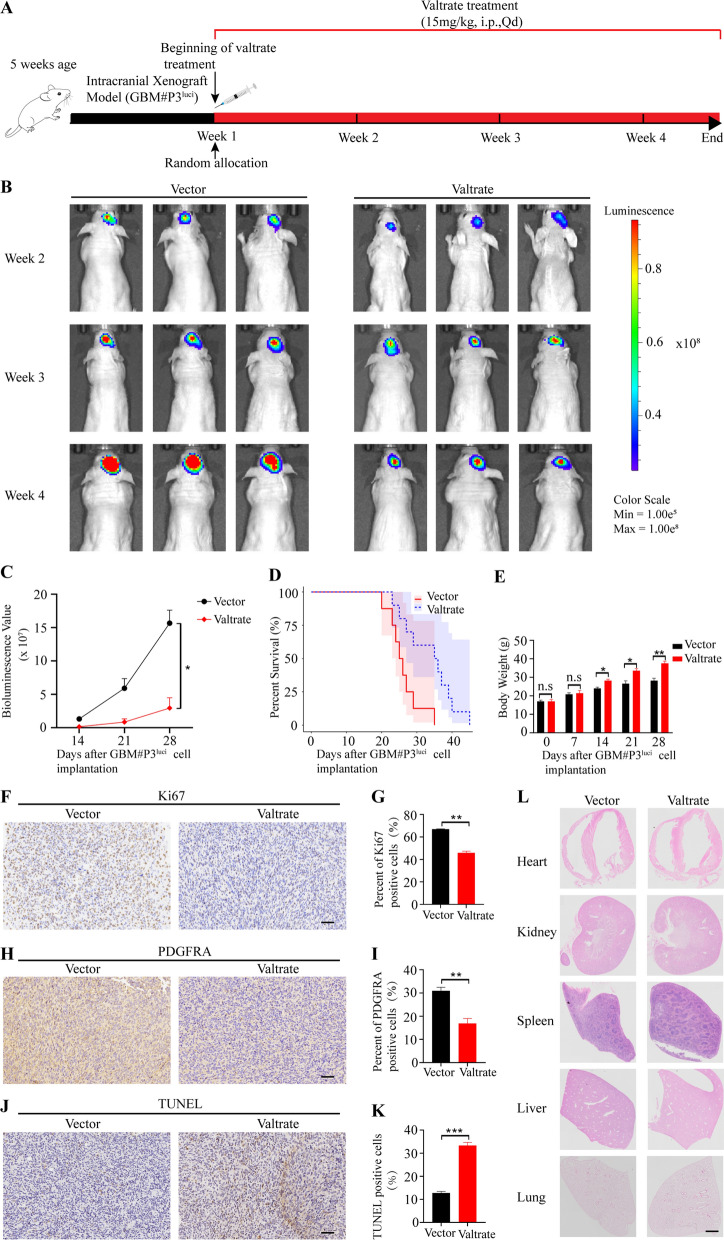
Valtrate inhibits tumor growth in an orthotopic xenograft model in nude mice. **A** Scheme (time line and protocol) for tumor cell implantation of luciferase expressing GBM#P3^luci^ cells and valtrate treatment in this study. **B** Bioluminescence imaging of GBM#P3^luci^ tumor-bearing mice. The bioluminescent signals were measured at the different time points post-treatment (weeks 2, 3, and 4 after tumor cell implantation). **C** Quantification of bioluminescence values to determine tumor growth after treatment at weeks 2, 3 and 4 in GBM#P3^luci^ tumor-bearing mice. **D** Kaplan–Meier analysis to assess overall survival of tumor-bearing nude mice under valtrate treatment relative to controls. The log-rank test was used to assess the statistical significance of the differences. **E** Quantification of the weight of tumor-bearing nude mice under valtrate treatment relative to controls. **F**–**I** Immunohistochemistry to detect Ki67 and PDGFRA in sections from xenografts from valtrate-treated animals and controls. Scale bar, 50 μm. **J**–**K** TUNEL assay to detect cell apoptosis in sections from xenografts from valtrate-treated animals and controls. Scale bar, 50 μm. **L** H&E staining of sections from major organs harvested from tumor-bearing animals treated with valtrate and controls. Scale bar, 50 μm. Data are shown as the mean ± SD and the differences between groups were analyzed with the Student’s *t*-test. *p < 0.05, **p < 0.01, ***p < 0.001. *i.p.* intraperitoneal. *Qd* once a day

To determine the potential therapeutic efficacy of valtrate in vivo, we treated nude mice orthotopically implanted with GBM#P3^luci^ cells overexpressing the luciferase reporter gene and assessed tumor growth through in vivo bioluminescence imaging. The experimental scheme (implantation and treatment schedule) is shown in Fig. 6A. The volumes of GBM xenografts in the nude mice treated with valtrate were significantly reduced relative to tumors in the control group by day 28 after implantation (Fig. 6B–C, Additional file 1: Figure S6A–B). Valtrate treatment significantly prolonged the survival of tumor-bearing mice (day 27 vs day 36, control vs treated) and the body weight also did not decrease as rapidly in tumor-bearing mice treated with valtrate relative to the controls (Fig. 6D and E). Ki67 (a marker of cell proliferation) and PDGFRA expression levels were decreased as assessed in IHC staining of sections from the valtrate-treated tumors of nude mice relative to controls . In addition, IHC in sections from mice demonstrated that the protein expression of p-MEK1/2 (Ser221), p-ERK1/2 (Thr202/Tyr204) and Bcl2 was significantly reduced, whereas Bax was significantly increased after valtrate treatment (Additional file 1: Figure S6C–J). TUNEL-positive cells were also increased in valtrate-treated tumors relative to controls (Fig. 6J–K). The results further suggested that valtrate induced apoptosis through the PDGFRA/MEK/ERK signaling pathway in GBM in vivo. Finally, H&E staining of sections from major organs harvested from valtrate-treated animals showed no observable tissue damage indicating that valtrate was not toxic to nude mice in this time frame (Fig. 6L). Overall, these data indicated that valtrate crossed the blood–brain barrier and effectively inhibited GBM proliferation in vivo.

## Discussion

Valtrate, a major component of the iridoids of *Valeriana*, has the property of crossing the blood–brain barrier [4]. In this study, we demonstrated that valtrate possessed potent antitumor activity against GBM cells in vitro and in vivo. The drug not only inhibited the proliferation, invasion and migration of GBM cells, but it also induced apoptosis in a dose-dependent manner in these cells.

Programmed cell death or apoptosis is an important mechanism for all multicellular organisms to control cell proliferation, maintain tissue homeostasis, and remove harmful or unwanted cells [20, 21]. However, defects in the physiological mechanisms of apoptosis may lead to the development of different human diseases, such as cancer. Therefore, new antitumor drugs are typically assessed for their ability to induce apoptosis [22]. Mechanistically, there are two main core apoptotic signaling pathways in mammalian cells, the internal pathway regulated by mitochondria and the external pathway mediated by death receptors [22]. A common pathway of intrinsic apoptotic cell death involves the permeability of the outer mitochondrial membrane (MOMP) and the release of cytochrome C from the mitochondria. MOMP as well as the release of cytochrome C into the cytoplasm is necessary to trigger apoptosis. Cytochrome C released from mitochondria is positively regulated by pro-apoptotic Bcl-2 family members, such as Bax, Bak, Bim, and Bid, and negatively regulated by anti-apoptotic Bcl-2 family members, such as Bcl-2, Bcl-xL, and MCL1 [23, 24]. The extrinsic pathway is activated by membrane death receptors such as tumor necrosis factor (TNF) receptor 1/2, Fas and the TNF-related apoptosis-inducing ligand receptors DR4 and DR5. The recruitment of binding proteins by these death receptors and activation of the apoptosis-initiating factors, including caspase-8 and -10, form the death-inducing signaling complex [25, 26]. Our results indicated that the mitochondrial membrane potential was reduced and mitochondrial function was impaired in GBM cells exposed to valtrate. We furthermore demonstrated that valtrate induced caspase-dependent apoptosis in GBM cell lines, which was accompanied by changes in Bcl-2, Bcl-xL and Bax proteins, and enhanced cleavage of C-PARP and C-caspases. Therefore, we believe that the anti-GBM activity of valtrate is exerted through the intrinsic apoptotic pathway. Hence, valtrate might be used as a candidate molecule derived from a natural product to improve GBM treatment.

One of the clinical features of GBM is the extensive infiltration of tumor cells into the brain paremchyma [27]. Like immature neurons and stem cells, glioma cells follow the same tortuous extracellular migration route, using blood vessels as guides. This infiltrative growth makes complete surgical excision impossible and increases the difficulty of chemo- and radiation therapy [28]. In contrast to benign tumors of the brain, GBM cells extensively infiltrate the tumor environment and imaging does not truly reflect the boundaries of the tumor. Thus, for GBM, a targeted “search & destroy” strategy may be more effective [29]. Therefore, the efficacy of a new drug for the treatment of GBM may depend in large part on whether it inhibits tumor invasion and migration. Our results demonstrated that valtrate led to cytoskeletal rearrangements and changes in expression of EMT-related proteins. Therefore, valtrate may block cell migration and invasion by inhibiting EMT.

PDGFRA, a transmembrane tyrosine kinase receptor, is an important anti-apoptotic molecule and peptide growth factor. PDGFRA and its major ligand PDGFA are key regulators of glial cell proliferation and found mainly in oligodendrocytes in the brain. PDGFRA is highly expressed in GBM and the overexpression of PDGFRA has been found to induce the development of GBM in animal models [19, 30–32]. PDGFA/PDGFRA signaling has been shown to induce EMT through stimulation of ZEB1, thereby promoting glioma tumor growth and invasion and GSCS stemness [16]. Furthermore, Gai et al. suggested that the combination of inhibitors targeting PDGFRA and another receptor mediating PDGFA signaling, EPHA2, represents a promising therapeutic strategy for GBM treatment [33]. Through RNA sequencing analysis, we found that *PDGFRA* was one of the most highly differentially downregulated genes in both valtrate-treated U251 and GBM#P3 cells. We then demonstrated that overexpression of PDGFRA partially rescued cells from the anti-GBM activity of valtrate. Therefore, downregulation of PDGFRA might be the mechanism underlying valtrate-induced inhibition of GBM.

**Fig. 7:**
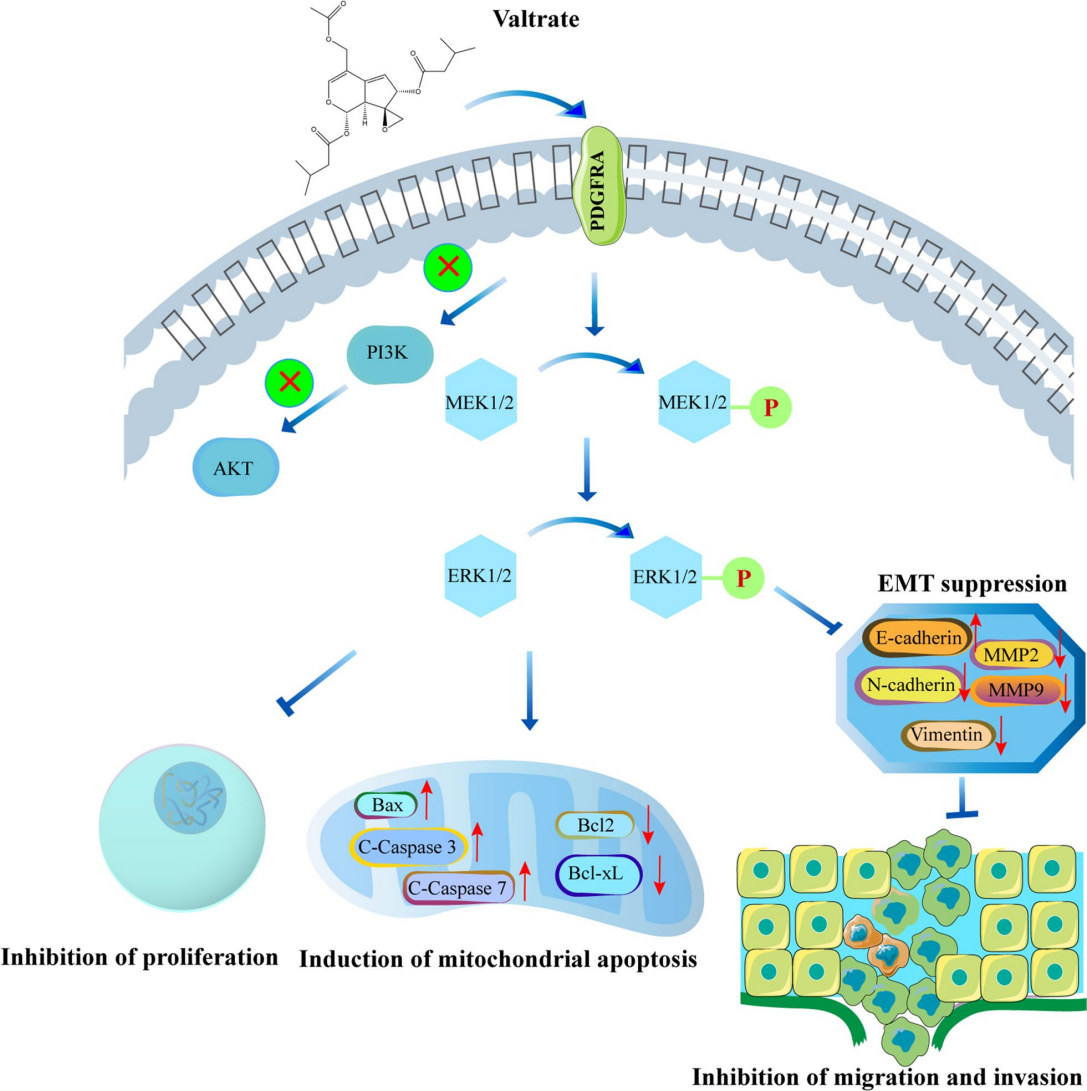
Valtrate inhibits GBM growth through the downregulation of PDGFRA. Scheme showing the central role of valtrate in the induction of mitochondrial apoptosis and the inhibition of cell migration and invasion in GBM through downregulation of PDGFRA

Because valtrate led to decreased levels of PDGFRA in GBM cells, we also investigated the levels of proteins mediating PDGFRA signaling in response to valtrate. PDGFA-activated PDGFRA triggers downstream signaling pathways, including MAPK, PI3-kinase/AKT and JAK/STAT, that play critical roles in cell proliferation, differentiation, invasion and migration [34]. PI3K/Akt/ mechanistic target of rapamycin (mTOR) and Ras/Raf/MEK/ERK signaling pathways are considered to be promising targets for cancer therapy [35]. Activated AKT, which is observed in ~ 70% of GBM patients, particularly in GBM with PTEN loss, and the disruption of RTK/PI3K signaling, are considered to be some of the major mechanisms driving GBM tumorigenesis and progression [36, 37]. The RAF/MEK/ERK signaling pathway is another MAPK pathway that promotes cell proliferation and survival, which has also been identified as a promising therapeutic target for cancer therapy [35]. The three isoforms of RAF, ARAF, BRAF and CRAF, and their downstream kinases, MEK1/2 and ERK1/2, constitute a coherent and coordinated signaling module that is involved in a range of physiological functions [17]. A multikinase inhibitor, sorafenib, inhibited proliferation and induced apoptosis in hepatocellular carcinoma cell lines, by inhibiting the MEK/ERK signaling pathway [38]. In our study, we found downregulation of PDGFRA, MEK1/2 phosphorylation and ERK1/2 phosphorylation in all GBM cells treated with valtrate. However, we did not detect any significant association between PI3K phosphorylation or AKT phosphorylation and the expression of PDGFRA in valtrate-treated GBM cells. In addition, tBHQ, a pharmacological agonist of ERK, reversed the effects of valtrate on GBM cell apoptosis, migration and invasion. These results all together indicated that the mechanism of the anti-GBM activity of valtrate is inhibition of the PDGFRA/MEK/ERK signaling pathway (Fig. 7).

Moreover, as a heterogeneous malignancy, GBM exhibits metabolic reprogramming through a variety of energy sources. Tumor cells mainly consume glucose to maintain anabolism and catabolism. Although aerobic glycolysis (Warburg effect) is less efficient for energy production, it is an effective pathway for driving the biosynthesis of key molecules required for uncontrolled cell growth and resistance to cell death [39–41]. Signaling pathways of oncogenes and tumor suppressors have been shown to play direct roles in facilitating the conversion of energy metabolism to aerobic glycolysis [42, 43]. Among the various signaling pathways that respond to oncogenic mutagenic events and regulate proliferation and apoptosis as well as aerobic glycolysis are members of the MAPK family, including the ERKs, the JNKs, and the p38 kinases [44–46]. Just as targeting cancer cell metabolism as a strategy to identify new therapeutic approaches offers a unique opportunity for drug discovery [40], and based on the fact that valtrate interferes with the MEK/ERK signaling pathway, it may be interesting to investigate the effects of valtrate on GBM cell metabolism to gain a more comprehensive understanding of the biological effects of valtrate, such as inducing apoptosis by interfering with the Warburg effect or disrupting temozolomide resistance, to prevent GBM recurrence.

In the present study, we did not explore the molecular mechanisms mediating the reduced levels of *PDGFRA* mRNA due to valtrate. Previous studies in many biological systems have demonstrated that the highly strained 3-membered ring of epoxides underlie their high reactivity, making them easy targets for ring opening by nucleophilic groups, such as amines, alcohols and sulfur [47, 48]. Epoxides produce biological activities through covalent binding to proteins or DNA [49, 50]. Therefore, valtrate, as a naturally-derived epoxide, is likely to covalently bind to its cellular targets to produce anti-GBM effects. One possibility is that valtrate may bind to key transcription factors regulating *PDGFRA* RNA and thus negatively affect transcription of the gene. In addition, we plan to further study the molecular structure of valtrate and its degradation products to gain a more comprehensive understanding of the basis for its antitumor activity. Moreover, larger scale animal experiments are needed to evaluate the efficacy and safety of valtrate to provide support for preclinical drug studies.

## Conclusions

In summary, we demonstrated that valtrate has antitumor activity in GBM in vitro and in vivo, and the anti-GBM activity is largely due to inhibition of the PDGFRA/MEK/ERK signaling pathway. Therefore, valtrate is a candidate drug for the treatment of GBM.

## Supplementary Information

**Additional file 1: Figure S1.** Valtrate inhibits cell proliferation in GBM cells. **Figure S2.** Valtrate promotes apoptosis in GBM cells via the mitochondrial pathway. **Figure S3.** Valtrate suppresses migration and invasion of GBM cells. **Figure S4.** PDGFRA is a potential target downregulated by valtrate in GBM cells. (A) Volcano plot showing the up- and downregulated genes, red and blue colors, respectively, obtained from RNA-seq analysis. Cells were treated with valtrate (U251: 2 μM, GBM#P3: 0.5 μM) for 48 h and RNA was isolated and sequenced. (B) Cell viability of LN229-PDGFRA-OE under the conditions indicated as determined with the CCK-8 assay. (C) Representative images of EdU assays for U251- and LN229-PDGFRA-OE cells under the conditions indicated. Scale bar, 50 μm. (D) Flow cytometry to detect the percentage of apoptotic U251- and GBM#P3-PDGFRA-OE cells under the conditions indicated as determined with annexin V-FITC and PI staining. (E) Representative images of 3D invasion assay for U251- and GBM#P3-PDGFRA-OE PDGFRA cells under the conditions indicated, with or without valtrate. Scale bar, 200 μm. All data are expressed as the mean ± SD of values from triplicate experiments and the differences between groups were analyzed with the Student’s *t*-test. *p < 0.05. **Figure S5.** Valtrate elicits anti-GBM activity through inhibition of the PDGFRA/MEK/ERK signaling pathway. **Figure S6.** Valtrate exerts its antitumor effects in vivo.

## Data Availability

The datasets used and/or analyzed during the current study are available from the corresponding author upon request.

## Abbreviations

GBM: Glioblastoma
EMT: Epithelial-mesenchymal transition
NHA: Normal human astrocyte
GFP: Green fluorescent protein
GSCS: Glioma stem cells
DMEM: Dulbecco’s Modified Eagle Medium
FBS: Fetal bovine serum
DMSO: Dimethylsulfoxide
TEM: Transmission electron microscopy
PBS: Phosphate-buffered saline
TBST: TBS *supplemented with 0.1% Tween‑20*
WB: Western Blot
IF: Immunofluorescence
IHC: Immunohistochemistry
H&E: Hematoxylin and eosin staining
PDGFRA: Platelet-derived growth factor receptor A
FN1: Fibronectin-1
GO: Gene ontology
KEGG: Kyoto Encyclopedia of Genes and Genomes
GEPIA: Gene Expression Profiling Interactive Analysis
Bcl-2: B-cell lymphoma 2
Bax: Bcl-2-associated x protein
MAPK: Mitogen-activated protein kinase
PI3K: Phosphatidylinositol-3-kinase
ERK: Extracellular signal-regulated kinase
BSA: Bovine serum albumin
DAPI: 2-(4-Amidinophenyl)-6-indolecarbamidine dihydrochloride
tBHQ: T-butylhydroquinone
mTOR: Mechanistic target of rapamycin
TNF-α: Tumor necrosis factor-alpha

## References

[1] Wei YT, Lu CF, Zhou P, Zhao L, Lyu X (2021). EIF4A3-induced circular RNA ASAP1 promotes tumorigenesis and temozolomide resistance of glioblastoma via NRAS/MEK1/ERK1–2 signaling. Neuro Oncol.

[2] Lu CF, Wei YT, Wang XF, Zhang ZR, Yin JX (2020). DNA-methylation-mediated activating of lncRNA SNHG12 promotes temozolomide resistance in glioblastoma. Mol Cancer.

[3] Newman DJ, Cragg GM (2020). Natural products as sources of new drugs over the nearly four decades from 01/1981 to 09/2019. J Nat Prod.

[4] Jugran AK, Rawat S, Bhatt ID, Rawal RS (2019). *Valeriana jatamansi*: an herbaceous plant with multiple medicinal uses. Phytother Res.

[5] Shi SN, Shi JL, Liu Y, Wang YL, Wang CG (2014). The anxiolytic effects of valtrate in rats involves changes of corticosterone levels. Evid Based Complement Alternat Med.

[6] Jugran AK, Bahukhandi A, Dhyani P, Bhatt ID, Rawal RS, Nandi SK (2016). Impact of altitudes and habitats on valerenic acid, total phenolics, flavonoids, tannins, and antioxidant activity of *Valeriana jatamansi*. Appl Biochem Biotechnol.

[7] Chen LP, Feng D, Qian YF, Cheng X, Song HZ (2021). Valtrate as a novel therapeutic agent exhibits potent anti-pancreatic cancer activity by inhibiting Stat3 signaling. Phytomedicine.

[8] Tian SS, Wang ZZ, Wu ZQ, Wei YY, Yang B, Lou SY (2020). Valtrate from Valeriana jatamansi Jones induces apoptosis and inhibits migration of human breast cancer cells *in vitro*. Nat Prod Res.

[9] Li XG, Chen T, Lin S, Zhao J, Chen PZ (2013). Valeriana jatamansi constituent IVHD-valtrate as a novel therapeutic agent to human ovarian cancer: in vitro and in vivo activities and mechanisms. Curr Cancer Drug Targets.

[10] Wang JW, Qi QC, Zhou WJ, Feng ZC, Huang B (2018). Inhibition of glioma growth by flavokawain B is mediated through endoplasmic reticulum stress induced autophagy. Autophagy.

[11] Kong Y, Feng ZC, Chen AJ, Qi QC, Han MZ (2019). The natural flavonoid galangin elicits apoptosis, pyroptosis, and autophagy in glioblastoma. Front Oncol.

[12] Zhou WJ, Wang JW, Qi QC, Feng ZC, Huang B (2018). Matrine induces senescence of human glioblastoma cells through suppression of the IGF1/PI3K/AKT/p27 signaling pathway. Cancer Med.

[13] Bjerkvig R, Laerum OD, Mella O (1986). Glioma cell interactions with fetal rat brain aggregates in vitro and with brain tissue in vivo. Cancer Res.

[14] Hu YT, Zhou WJ, Xue ZY, Liu XM, Feng ZC (2022). Thiabendazole inhibits glioblastoma cell proliferation and invasion targeting mini-chromosome maintenance protein 2. J Pharmacol Exp Ther.

[15] Ye QH, Zhu WW, Zhang JB (2016). GOLM1 modulates EGFR/RTK cell-surface recycling to drive hepatocellular carcinoma metastasis. Cancer Cell.

[16] Zhang L, Zhang W, Li Y (2016). SHP-2-upregulated ZEB1 is important for PDGFRα-driven glioma epithelial - mesenchymal transition and invasion in mice and humans. Oncogene.

[17] Ullah R, Yin Q, Snell AH, Wan L (2022). RAF-MEK-ERK pathway in cancer evolution and treatment. Semin Cancer Biol..

[18] Yang Q, Jiang W, Hou P (2019). Emerging role of PI3K/AKT in tumor-related epigenetic regulation. Semin Cancer Biol.

[19] Xu R, Ji JX, Zhang X, Han MZ, Zhang C (2017). PDGFA/ PDGFRα- regulated GOLM1 promotes human glioma progression through activation of AKT. J Exp Clin Cancer Res.

[20] Goldar S, Khaniani MS, Derakhshan SM, Baradaran B (2015). Molecular mechanisms of apoptosis and roles in cancer development and treatment. Asian Pac J Cancer Prev.

[21] Sankari SL, Masthan KM, Babu NA, Bhattacharjee T, Elumalai M (2012). Apoptosis in cancer—an update. Asian Pac J Cancer Prev.

[22] Carneiro BA, El-Deiry WS (2020). Targeting apoptosis in cancer therapy. Nat Rev Clin Oncol.

[23] Yang J, Liu X, Bhalla K, Kim CN, Ibrado AM (1997). Prevention of apoptosis by Bcl-2: release of cytochrome c from mitochondria blocked. Science.

[24] Bertheloot D, Latz E, Franklin BS (2021). Necroptosis, pyroptosis and apoptosis: an intricate game of cell death. Cell Mol Immunol.

[25] Micheau O, Tschopp J (2003). Induction of TNF receptor I-mediated apoptosis via two sequential signaling complexes. Cell.

[26] Muzio M, Chinnaiyan AM, Kischkel FC, O'Rourke K, Shevchenko A (1996). FLICE, a novel FADD-homologous ICE/CED-3-like protease, is recruited to the CD95 (Fas/APO-1) death–inducing signaling complex. Cell.

[27] Li C, Wang S, Yan JL, Torheim T, Boonzaier NR (2019). Characterizing tumor invasiveness of glioblastoma using multiparametric magnetic resonance imaging. J Neurosurg.

[28] Cuddapah VA, Robel S, Watkins S, Sontheimer H (2014). A neurocentric perspective on glioma invasion. Nat Rev Neurosci.

[29] Claes A, Idema AJ, Wesseling P (2007). Diffuse glioma growth: a guerilla war. Acta Neuropathol.

[30] Liu KW, Hu B, Cheng SY (2011). Platelet-derived growth factor receptor alpha in glioma: a bad seed. Chin J Cancer.

[31] Martinho O, Longatto-Filho A, Lambros MBK, Martins A, Pinheiro C (2009). Expression, mutation and copy number analysis of platelet-derived growth factor receptor A (PDGFRA) and its ligand PDGFA in gliomas. Br J Cancer.

[32] Jun HJ, Appleman VA, Wu HJ, Rose CM, Pineda JJ (2018). A PDGFRα-driven mouse model of glioblastoma reveals a stathmin1- mediated mechanism of sensitivity to vinblastine. Nat Commun.

[33] Gai QJ, Fu Z, He J, Mao M, Yao XX (2022). EPHA2 mediates PDGFA activity and functions together with PDGFRA as prognostic marker and therapeutic target in glioblastoma. Signal Transduct Target Ther.

[34] Blume-Jensen P, Hunter T (2001). Oncogenic kinase signaling. Nature.

[35] Asati V, Mahapatra DK, Bharti SK (2016). PI3K/Akt/mTOR and Ras/Raf/MEK/ERK signaling pathways inhibitors as anticancer agents: structural and pharmacological perspectives. Eur J Med Chem.

[36] Brennan CW, Verhaak RG, McKenna A, Campos B, Noushmehr H, Salama SR, Zheng S, Chakravarty D, Sanborn JZ, Berman SH (2013). The somatic genomic landscape of glioblastoma. Cell.

[37] Chin YR, Yuan X, Balk SP, Toker A (2014). PTEN-deficient tumors depend on AKT2 for maintenance and survival. Cancer Discov.

[38] Liu L, Cao YC, Chen C, Zhang XM, McNabola A (2006). Sorafenib blocks the RAF/MEK/ERK pathway, inhibits tumor angiogenesis, and induces tumor cell apoptosis in hepatocellular carcinoma model PLC/PRF/5. CancerRes.

[39] Nguyen TTT, Shang E, Westhoff MA, Karpel-Massler G, Siegelin MD (2022). Therapeutic drug-induced metabolic reprogramming in glioblastoma. Cells.

[40] Agnihotri S, Zadeh G (2016). Metabolic reprogramming in glioblastoma: the influence of cancer metabolism on epigenetics and unanswered questions. Neuro Oncol.

[41] Pavlova NN, Thompson CB (2016). The emerging hallmarks of cancer metabolism. Cell Metab.

[42] Hsu PP, Sabatini DM (2008). Cancer cell metabolism: Warburg and beyond. Cell.

[43] Jones RG, Thompson CB (2009). Tumor suppressors and cell metabolism: a recipe for cancer growth. Genes Dev.

[44] Cargnello M, Roux PP (2011). Activation and function of the MAPKs and their substrates, the MAPK-activated protein kinases. Microbiol Mol Biol Rev.

[45] Papa S, Choy PM, Bubici C (2019). The ERK and JNK pathways in the regulation of metabolic reprogramming. Oncogene.

[46] Mukhopadhyay S, Vander Heiden MG, McCormick F (2021). The metabolic landscape of RAS-driven cancers from biology to therapy. Nat Cancer.

[47] Gomes AR, Varela CL, Tavares-da-Silva EJ, Roleira FMF (2020). Epoxide containing molecules: a good or a bad drug design approach. Eur J Med Chem.

[48] Kaur B, Singh P (2022). Epoxides: developability as active pharmaceutical ingredients and biochemical probes. Bioorg Chem.

[49] Hughes TB, Miller GP, Swamidass SJ (2015). Modeling epoxidation of drug-like molecules with a deep machine learning network. ACS Cent Sci.

[50] Kalgutkar AS, Didiuk MT (2009). Structural alerts, reactive metabolites, and protein covalent binding: how reliable are these attributes as predictors of drug toxicity?. Chem Biodivers.

