# Supplementary material for: Valtrate, an iridoid compound in Valeriana, elicits anti-glioblastoma activity through inhibition of the PDGFRA/MEK/ERK signaling pathway
Source: J Transl Med. 2023 Feb 24;21:147. doi: 10.1186/s12967-023-03984-0 (PMC9960449; doi:10.1186/s12967-023-03984-0)
Supplement: Supplementary file 1 — Additional file 1: Figure S1. Valtrate inhibits cell proliferation in GBM cells. Figure S2. Valtrate promotes apoptosis in GBM cells via the mitochondrial pathway. Figure S3. Valtrate suppresses migration and invasion of GBM cells. Figure S4. PDGFRA is a potential target downregulated by valtrate in GBM cells. (A) Volcano plot showing the up- and downregulated genes, red and blue colors, respectively, obtained from RNA-seq analysis. Cells were treated with valtrate (U251: 2 μM, GBM#P3: 0.5 μM) for 48 h and RNA was isolated and sequenced. (B) Cell viability of LN229-PDGFRA-OE under the conditions indicated as determined with the CCK-8 assay. (C) Representative images of EdU assays for U251- and LN229-PDGFRA-OE cells under the conditions indicated. Scale bar, 50 μm. (D) Flow cytometry to detect the percentage of apoptotic U251- and GBM#P3-PDGFRA-OE cells under the conditions indicated as determined with annexin V-FITC and PI staining. (E) Representative images of 3D invasion assay for U251- and GBM#P3-PDGFRA-OE PDGFRA cells under the conditions indicated, with or without valtrate. Scale bar, 200 μm. All data are expressed as the mean ± SD of values from triplicate experiments and the differences between groups were analyzed with the Student’s t-test. *p < 0.05. Figure S5. Valtrate elicits anti-GBM activity through inhibition of the PDGFRA/MEK/ERK signaling pathway. Figure S6. Valtrate exerts its antitumor effects in vivo. [file 12967_2023_3984_MOESM1_ESM.doc]

**Additional file**


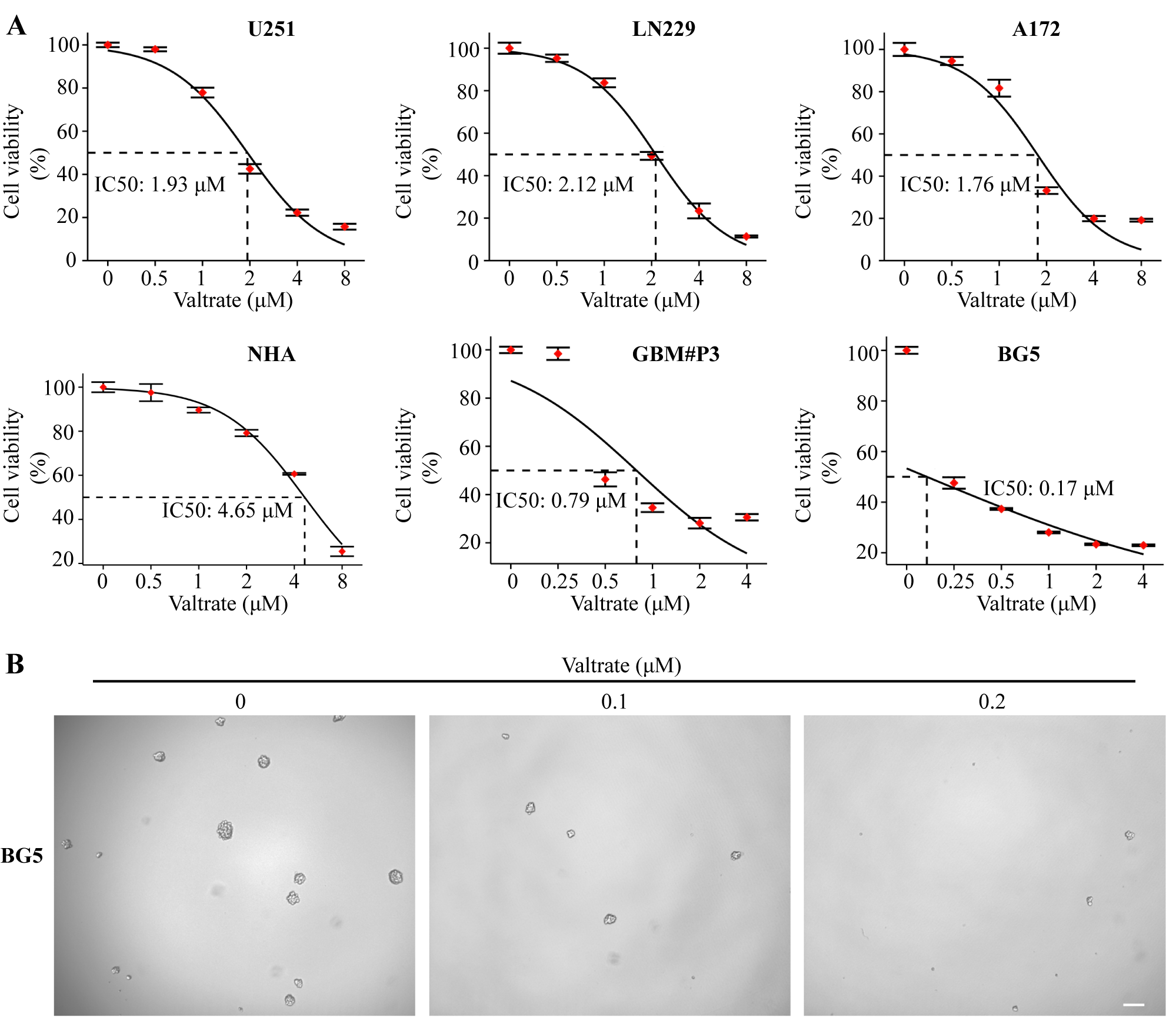


**Fig. S1. Valtrate inhibits cell proliferation in GBM cells.**

(A) IC50 values for U251, LN229, A172, GBM#P3, BG5 and NHA determined with the CCK-8 assay. (B) Representative images from tumorsphere formation assay for BG5 treated with valtrate. Scale bar = 50 μm.


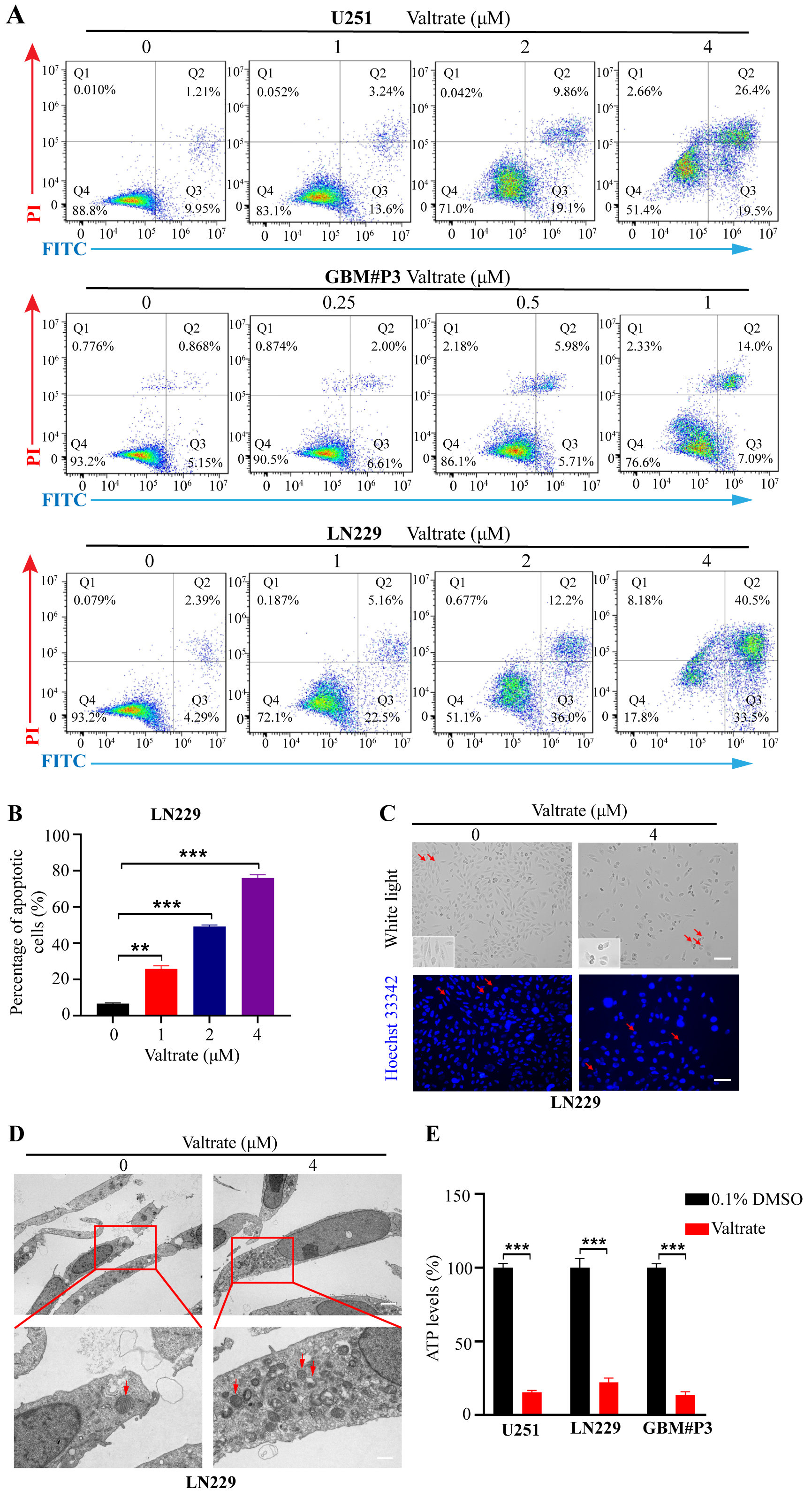


**Fig. S2. Valtrate promotes apoptosis in GBM cells via the mitochondrial pathway.**

(A) Flow cytometry to determine the percentage of apoptotic cells assessed with annexin V-FITC and PI double staining in U251, LN229 and GBM#P3 cells treated with valtrate for 48 h. (B) Quantification of annexin V-FITC and PI staining to detect apoptotic LN229 cells under treatment of valtrate for 48 h. (C) Representative images of the cellular morphology of valtrate-treated LN229 cells at 48 h, as observed under a phase contrast microscope. Enlarged images in the inset of LN229 cells with morphological changes highlighted by the red arrows (upper panel). Fluorescence imaging of valtrate-treated cells stained with Hoechst 33342 to examine nuclear morphology (lower panel). Scale bar, 50 μm. (D) Transmission electron microscopy to illuminate the mitochondrial ultrastructure of valtrate-treated LN229 cells. Scale bar, 5.0 μm; scale bar of the local enlargement, 2.0 μm. The red arrows highlight mitochondria. (E) Quantification of ATP levels detected in U251, LN229 and GBM#P3 treated with valtrate (4, 4 and 1 μM, respectively) for 48 h. ATP was detected with the ATP Detection Kit. Data are shown as the mean ± SD and the differences between groups were analyzed with the Student’s *t*-test. **p < 0.01, ***p < 0.001.


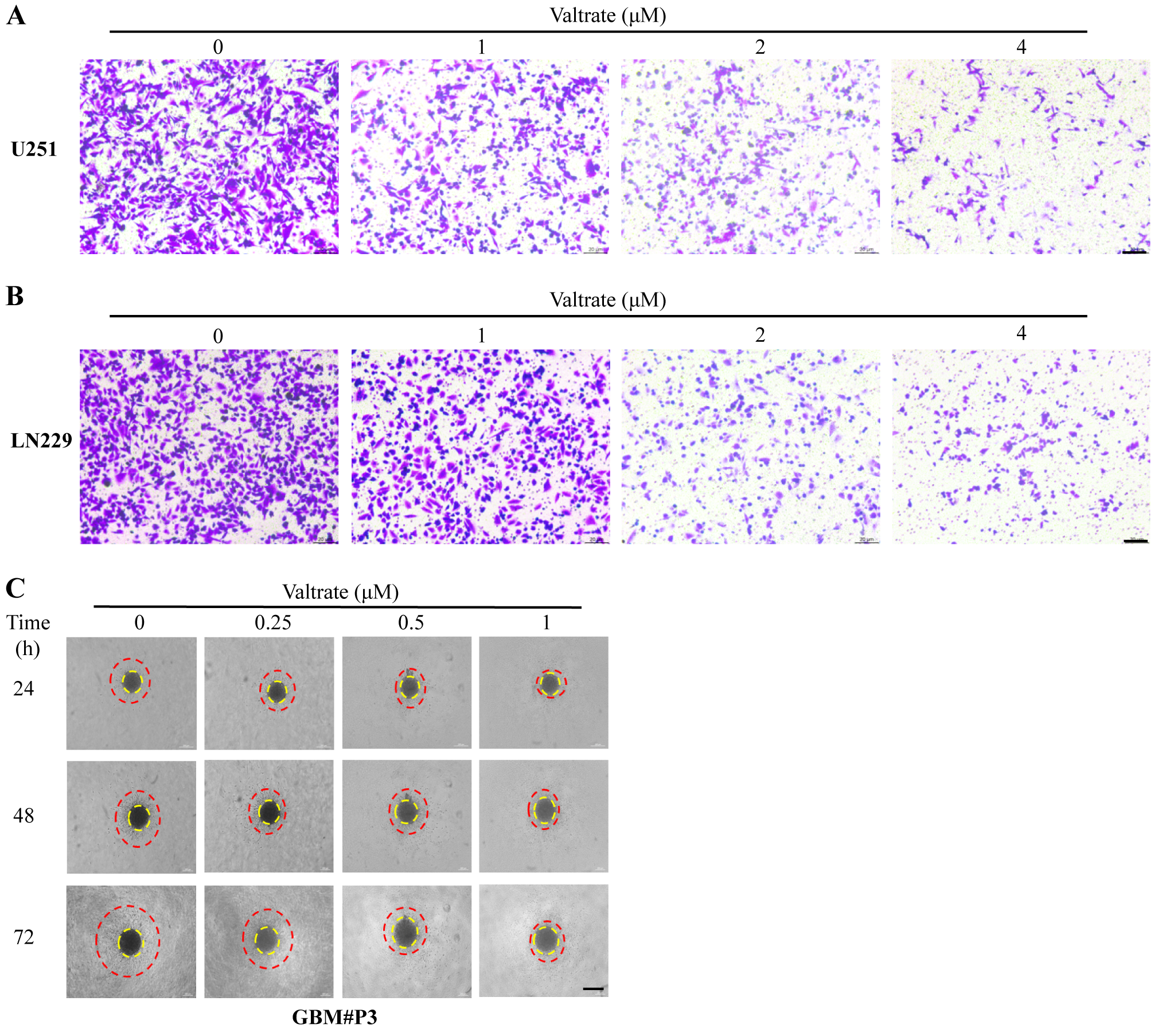


**Fig. S3.** **Valtrate suppresses migration and invasion of GBM cells.**

(A-B) Transwell migration assay of valtrate-treated U251 and LN229 cells and controls. Cells were fixed and stained with crystal violet. Scale bar, 20 μm. (C) Representative images of 3D invasion assay for GBM#P3 cells treated with different concentrations of valtrate at different time points. Scale bar, 200 μm.


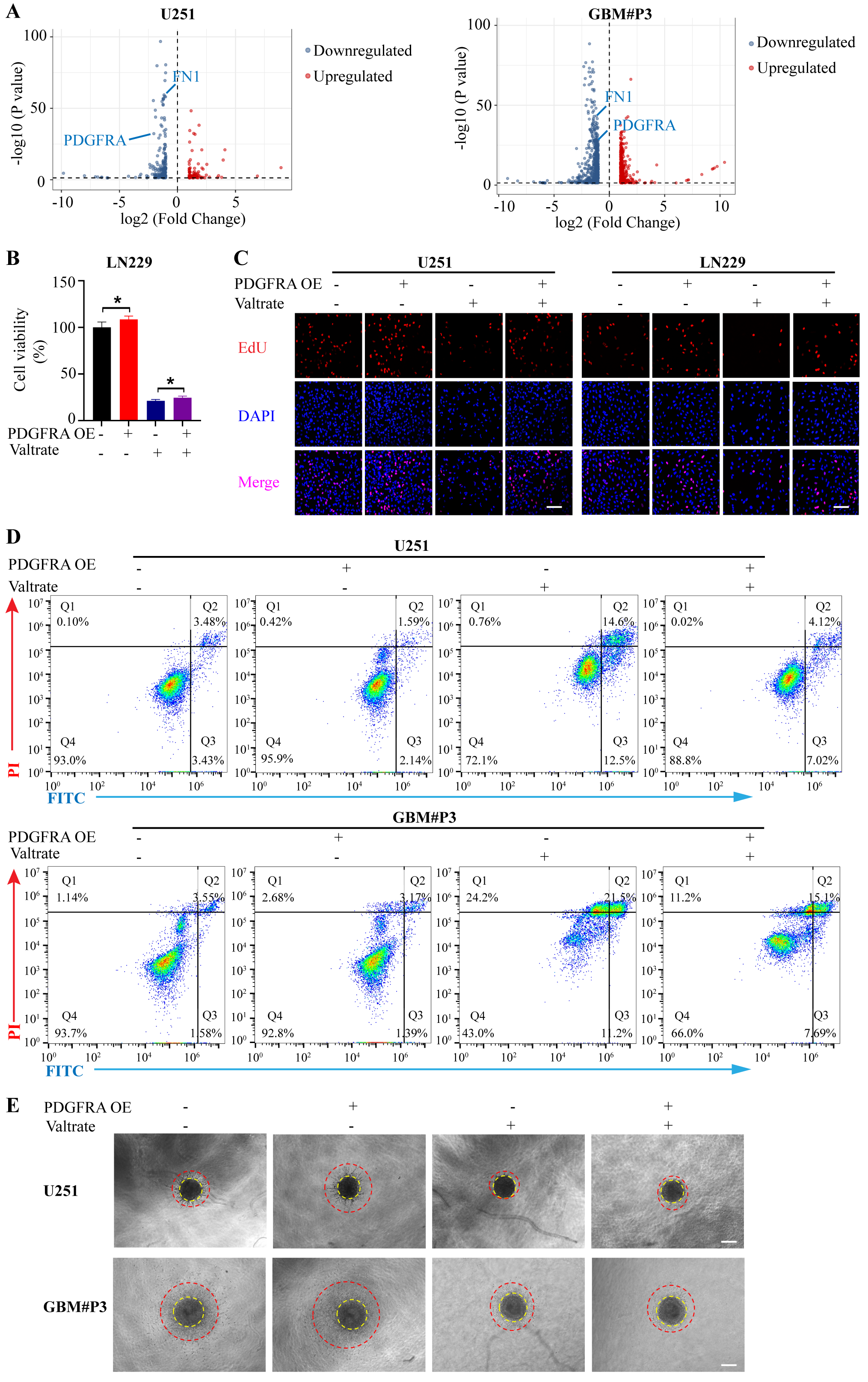


**Fig. S4. PDGFRA is a potential target downregulated by valtrate in GBM cells.** (A) Volcano plot showing the up- and downregulated genes, red and blue colors, respectively, obtained from RNA-seq analysis. Cells were treated with valtrate (U251: 2 μM, GBM#P3: 0.5 μM) for 48 h and RNA was isolated and sequenced. **(**B) Cell viability of LN229-PDGFRA-OE under the conditions indicated as determined with the CCK-8 assay. (C) Representative images of EdU assays for U251- and LN229-PDGFRA-OE cells under the conditions indicated. Scale bar, 50 μm. (D) Flow cytometry to detect the percentage of apoptotic U251- and GBM#P3-PDGFRA-OE cells under the conditions indicated as determined with annexin V-FITC and PI staining. (E) Representative images of 3D invasion assay for U251- and GBM#P3-PDGFRA-OE PDGFRA cells under the conditions indicated, with or without valtrate. Scale bar, 200 μm. All data are expressed as the mean ± SD of values from triplicate experiments and the differences between groups were analyzed with the Student’s *t*-test. *p < 0.05.


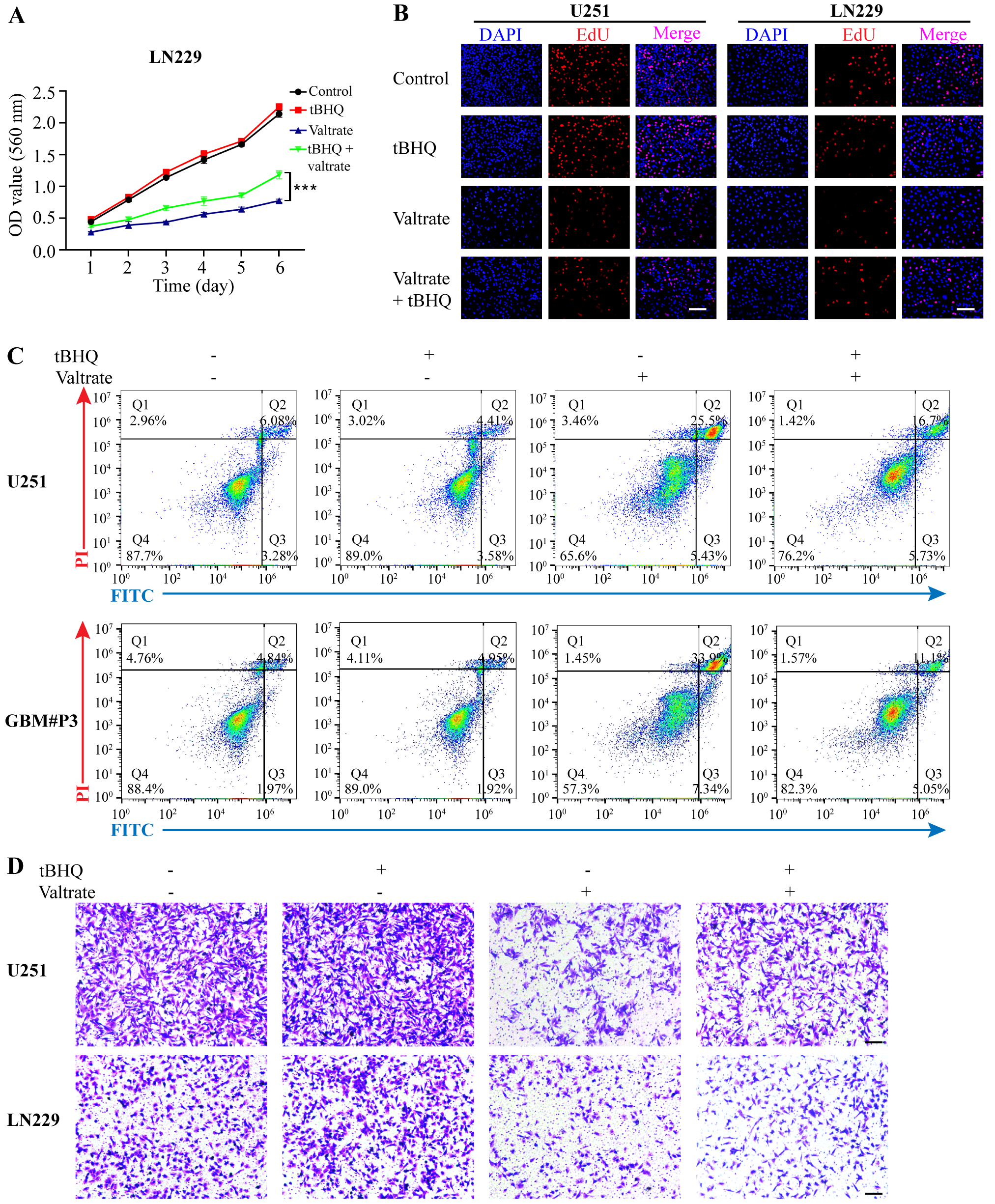


**Fig. S5. Valtrate elicits anti-GBM activity through inhibition of the PDGFRA/MEK/ERK signaling pathway.**

(A) Relative cell growth for valtrate-treated LN229 cells with or without tBHQ, as determined using OD values from the CCK-8 assay. LN229 cells were treated under the conditions indicated: DMSO (vehicle control), tBHQ, valtrate or valtrate + tBHQ. LN229: 2 μΜ valtrate and tBHQ: 50 μM. (B) Representative fluorescence images of EdU assays performed on valtrate-treated U251 and LN229 cells with or without tBHQ. U251 and LN229 cells were treated under the conditions indicated: DMSO (vehicle control), tBHQ, valtrate or valtrate + tBHQ. Scale bar, 50 μm. (C) Flow cytometry to detect apoptosis in U251 and GBM#P3 cells with annexin V-FITC and PI staining. U251 and GBM#P3 cells treated under the conditions indicated: DMSO, tBHQ, valtrate or valtrate + tBHQ. (D) Representative images of transwell assays for valtrated-treated U251 and LN229 cells in the presence of tBHQ. U251 and LN229 cells were treated under the conditions indicated: DMSO, tBHQ, valtrate or valtrate + tBHQ. Scale bar, 20 μm. Data are expressed as the mean ± SD of values from triplicate experiments and the differences between groups were analyzed with a Student’s *t*-test. ***p < 0.001.


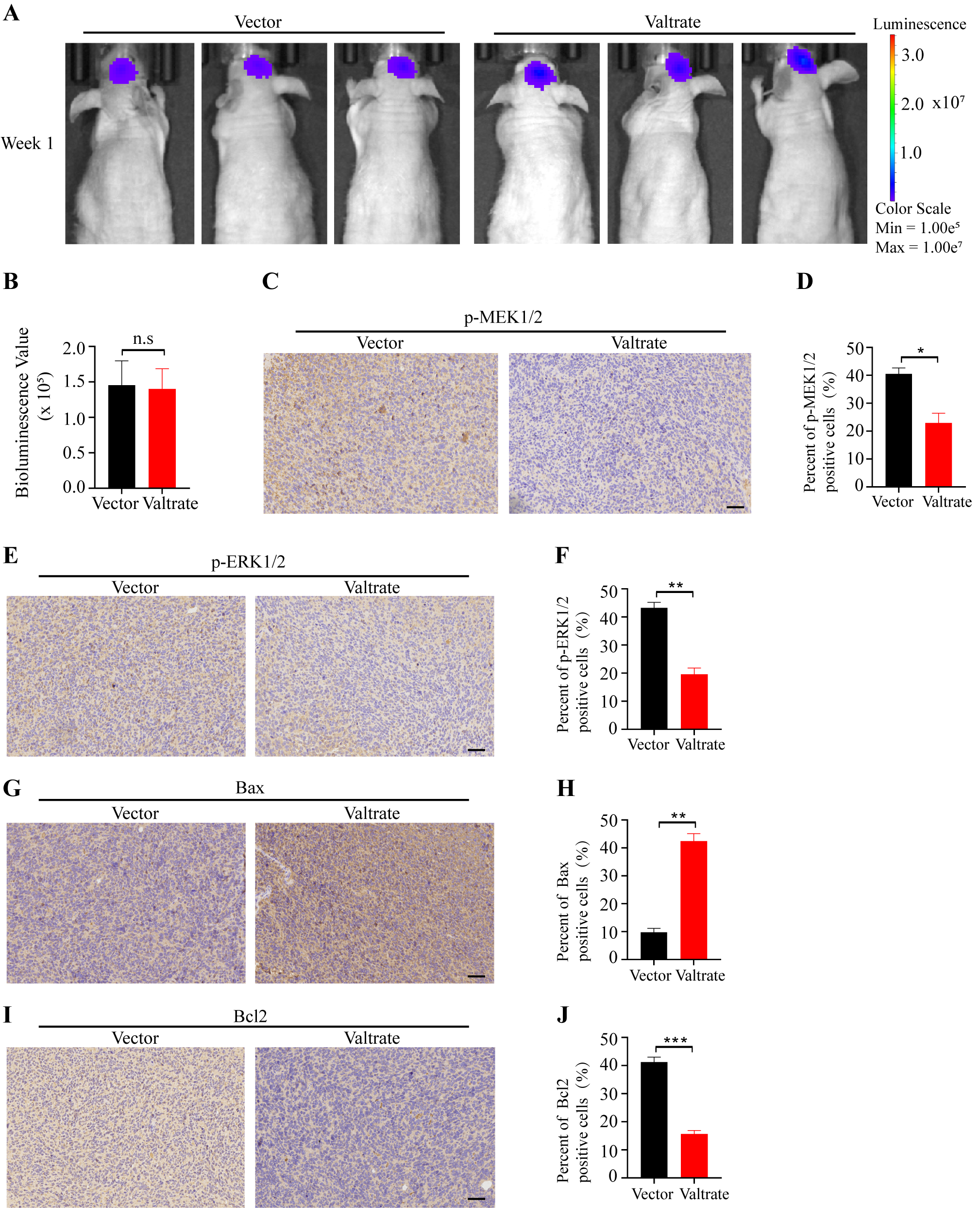


**Fig. S6.** **Valtrate exerts its antitumor effects in vivo.**

(A) Bioluminescent imaging of GBM#P3^luci^ orthotopic xenograft tumors at week 1 after implantation. (B) Quantification of the initial tumor bioluminescence values (week 1 after implantation). No statistical difference in the initial tumor volume between the vector and valtrate-treated groups (p > 0.05) was observed. (C-J) IHC to detect p-MEK1/2 (Ser221), p-ERK1/2 (Thr202/Tyr204), Bax and Bcl2 in sections from xenografts from valtrate-treated animals and controls, and quantitation of the results. Scale bar, 50 μm. All data are expressed as the mean ± SD of values from triplicate experiments and the differences between groups were analyzed by the Student’s *t*-test. n.s. = not significant, *p < 0.05, **p < 0.01, and ***p < 0.001.
